# Construction and validation of an angiogenesis-related gene expression signature associated with clinical outcome and tumor immune microenvironment in glioma

**DOI:** 10.3389/fgene.2022.934683

**Published:** 2022-08-11

**Authors:** Tianhao Hu, Yutao Wang, Xiaoliang Wang, Run Wang, Yifu Song, Li Zhang, Sheng Han

**Affiliations:** ^1^ Department of Neurosurgery, The First Hospital of China Medical University, Shenyang, China; ^2^ Department of Urology, The First Hospital of China Medical University, Shenyang, China; ^3^ Department of Neurosurgery, Huazhong University of Science and Technology Union Shenzhen Hospital, Shenzhen, China

**Keywords:** angiogenesis, glioma, gene signature, prognosis, tumor immune microenvironment

## Abstract

**Background:** Glioma is the most prevalent malignant intracranial tumor. Many studies have shown that angiogenesis plays a crucial role in glioma tumorigenesis, metastasis, and prognosis. In this study, we conducted a comprehensive analysis of angiogenesis-related genes (ARGs) in glioma.

**Methods:** RNA-sequencing data of glioma patients were obtained from TCGA and CGGA databases. V*ia* consensus clustering analysis, ARGs in the sequencing data were distinctly classified into two subgroups. We performed univariate Cox regression analysis to determine prognostic differentially expressed ARGs and least absolute shrinkage and selection operator Cox regression to construct a 14-ARG risk signature. The CIBERSORT algorithm was used to explore immune cell infiltration, and the ESTIMATE algorithm was applied to calculate immune and stromal scores.

**Results:** We found that the 14-ARG signature reflected the infiltration characteristics of different immune cells in the tumor immune microenvironment. Additionally, total tumor mutational burden increased significantly in the high-risk group. We combined the 14-ARG signature with patient clinicopathological data to construct a nomogram for predicting 1-, 3-, and 5-year overall survival with good accuracy. The predictive value of the prognostic model was verified in the CGGA cohort. *SPP1* was a potential biomarker of glioma risk and was involved in the proliferation, invasion, and angiogenesis of glioma cells.

**Conclusion:** In conclusion, we established and validated a novel ARG risk signature that independently predicted the clinical outcomes of glioma patients and was associated with the tumor immune microenvironment.

## Introduction

Glioma is the most common malignant tumor of the central nervous system (CNS), accounting for approximately 15% of all brain tumors (Ostrom et al., 2019). By degree of malignancy, gliomas are classified into low-grade gliomas (LGGs) and glioblastoma multiforme (GBM) (Louis et al., 2016). Despite the availability of a variety of treatment options including surgery, radiotherapy and chemotherapy, immunotherapy, and targeted therapy (Aldape et al., 2019), prognosis in glioma has remained poor; this is especially true in GBM patients, whose median survival time is < 15 months (Chen et al., 2017; Xu et al., 2020a). This poor prognosis is largely attributed to aberrant angiogenesis, high invasiveness, and therapeutic resistance (Furnari et al., 2007; Tan et al., 2018). According to previous research, gliomas with *IDH* mutation and 1p/19q codeletion have a relatively favorable prognosis (Eckel-Passow et al., 2015). The methylation status of the MGMT promoter has emerged as a key predictive biomarker of glioma and a potential predictor of response to temozolomide (Wick et al., 2014; Butler et al., 2020). However, additional research is needed to explore novel prognostic biomarkers and identify new therapeutic targets.

Angiogenesis refers to the formation of new blood vessels in the existing vasculature, which plays a pivotal role in many physiological and pathological processes such as embryonic development, wound healing, and tumor progression (Carmeliet, 2005). The pathophysiological processes of angiogenesis are reported to play critical roles in glioma development and therapeutic resistance (Onishi et al., 2011). Due to the important role of angiogenesis in gliomas, the use of angiogenesis-related genes (ARGs) to effectively stratify risk determining potential targets for individualized treatment is a promising research strategy. However, there have been few studies on the link between ARGs and prognosis in patients with glioma.

More recently, numerous studies have shown that the tumor immune microenvironment (TIME) plays a critical role in tumor progression and response to therapeutics (Quail and Joyce, 2017). Tumor-infiltrating immune cells can regulate tumor growth and invasion and are key components of the tumor microenvironment (TME) (Xu et al., 2020a; Xu Y. et al., 2020c). `The existing body of research on the TME suggests that immunotherapy is a promising method for the treatment of malignant tumors (Kruger et al., 2019; Xu et al., 2020b). In addition, the components of the TIME are closely correlated with the efficacy of immunotherapy.

**FIGURE 1:**
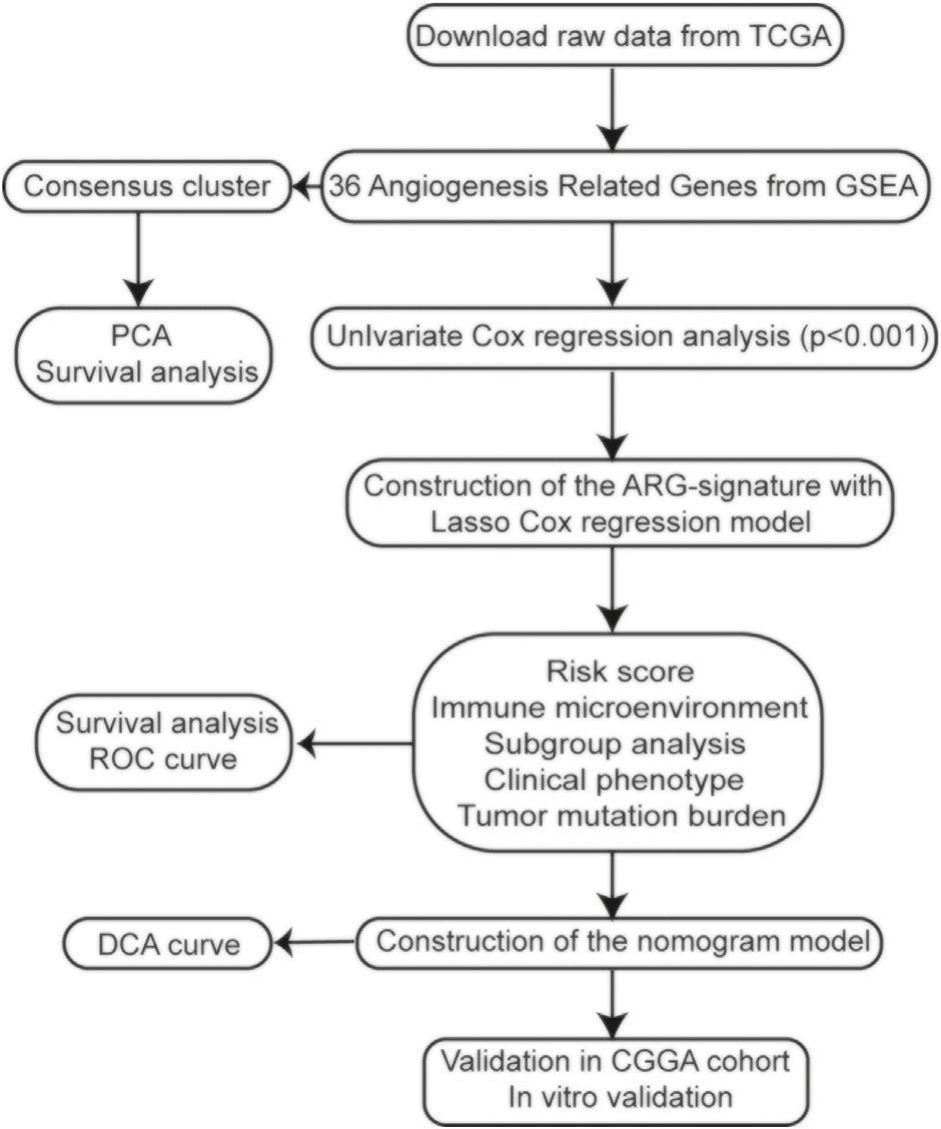
Flow chart of the study.

In this study, we used data from the Cancer Genome Atlas (TCGA) and the Chinese Glioma Genome Atlas (CGGA) databases to explore the expression profiles and prognostic value of ARGs in gliomas. Then, based on ARG expression, we constructed clustering subgroups and risk models to verify the predictive value of ARGs in risk stratification and clinical outcome. We also evaluated the associations between the ARG expression risk signature and the immune microenvironment, tumor mutational burden (TMB), and immunotherapy response. Finally, to validate the clinical application of the ARG expression signature, a nomogram model was developed to predict the overall survival (OS) rates of glioma patients. The flow chart of this study is shown in Figure 1.

## Materials and methods

### Data resources

The TCGA dataset provided raw counts of RNA-sequencing data (FPKM values) and accompanying clinical information for glioma samples. The expression data and clinical information of the validation RNA-seq cohort CGGA693 were acquired from the CGGA website. We transformed the FPKM values into transcript per million (TPM) values (Wagner et al., 2012); all values of the expression data were log_2_ (*x* + 1)-transformed. The characteristics of patients in the TCGA and CGGA cohorts are summarized in Supplementary Table S1.

### Consensus clustering analysis

We used the R package ConsensusClusterPlus, version 1.54.0, for consistency analysis. The maximum number of clusters was 6, and 80% of the total sample was drawn 100 times, clusterAlg = “hc,” innerLinkage = 'ward.D2.’ CDF and consensus matrices were used to calculate the appropriate number of subtypes. Then, we used PCA to detect differential gene expression between the two subtypes.

### Construction of the angiogenesis-related gene signature

Univariate Cox regression analysis was performed to screen out ARGs significantly correlated with survival (*p* < 0.001). Next, biomarkers of the 28 ARGs were identified from the LASSO Cox regression algorithm using the glmnet package in R. We calculated the risk score of each glioma patient by the following formula:

$$Riskscore=\sum_{i=1}^{n}\left(Coef_{i}*x_{i}\right),$$

where *Coef*_*i*_ is the coefficient of each ARG and *x*_*i*_ is the expression level of each ARG. In the risk score model, samples were subdivided into high- and low-risk groups according to the median risk score value.

### Tumor-infiltrating immune microenvironment analysis

CIBERSORT is a deconvolution method for expression matrices of immune cell subsets (Newman et al., 2019). LM22 is a gene signature matrix that specifies the content of immune cell types. We used the CIBERSORT package in R to calculate the number of immune cells per sample, setting the permutation to 1,000 and selecting *p* < 0.05 as the screening threshold. The ESTIMATE algorithm was used to evaluate immune score, tumor purity, and stromal score (Yoshihara et al., 2013). We calculated abundances of immune infiltrates, including B cells, CD4^+^ T cells, CD8^+^ T cells, neutrophils, macrophages, and dendritic cells (DCs), using Tumor IMmune Estimation Resource (TIMER) (Li et al., 2017).

### Single-sample gene set enrichment analysis

We used the ssGSEA method with the Gene Set Variation Analysis (GSVA) package in R to evaluate infiltration levels of different immune cells, the related expression pathways, and the activity of immune-related functions.

### Tumor mutational burden analysis

We used the Maftools package to analyze and visualize somatic-mutation data in order to study the mutational landscapes of glioma patients (Mayakonda et al., 2018). TMB was defined as the total number of somatic mutations per million bases.

### Survival analysis

We conducted Kaplan–Meier (KM) analysis to characterize the differences in survival of glioma patients using the R packages survival and survminer. The significance of differences in survival time was determined by using the log-rank test (*p* < 0.05).

### Building and verification of the nomogram

The nomogram was constructed using the rms package in R. We created a calibration curve to examine the consistency between the actual survival rate and expected survival rate. We built the nomogram model based on our multivariate Cox regression results. We created calibration plots of the nomogram for 1-, 3-, and 5-year OS using the “calibrate” function in rms. Decision curve analysis (DCA) was used to assess the clinical net benefit.

### Protein–protein interaction

The protein–protein interaction (PPI) analysis of ARGs was performed by using the STRING website (https://www.string-db.org/). The interaction analysis was conducted by Cytoscape software. The hub nodes were identified by the MCC method of cytoHubba plugin.

### Cell culture

We cultivated the glioma cell lines U87 and LN229 in high-glucose Dulbecco’s modified Eagle’s medium (DMEM) with 10% fetal bovine serum (FBS), 100 U/ml penicillin, and 100 μg/ml streptomycin at 37°C with 5% CO_2_. *SPP1* small-interfering RNA (siRNA) sequences were as follows: si-*SPP1*-1: CCA​GTT​AAA​CAG​GCT​GAT​T; si-*SPP1*-2: GTC​TCA​CCA​TTC​TGA​TGA​A.

### Western blotting

Western blot (WB) analysis was performed as previously reported (Han et al., 2017). Briefly, we extracted total proteins using a Total Cell Protein Extraction Kit (KeyGen Biotechnology, Nanjing, China). Equal amounts of protein were electrophoresed, transferred onto nitrocellulose membranes, and blocked with 2% bovine serum albumin (BSA). We used primary antibodies against *SPP1* (1:1,000; ab69498; Abcam, Cambridge, United Kingdom) to detect the expression of this protein. After washing them four times with Tris-buffered saline + Polysorbate 20 (TBST)/0.1% Tween-20, we incubated the membranes with the corresponding secondary antibody. A glyceraldehyde 3-phosphate dehydrogenase (GAPDH) protein band was used as a control to normalize protein levels. We visualized protein bands using a chemiluminescence kit (Beyotime Biotechnology, Beijing, China).

### Cell viability assay

We inoculated the treated U87 and LN229 cells in 96-well plates at a density of 1 × 10^3^ cells/well for 24, 48, 72, 96, and 120 h. The plates were examined using a cell viability assay kit (Promega Corp., Fitchburg, WI, United States) in accordance with the manufacturer’s protocol, as described previously (Wang et al., 2021).

### 5-ethynyl-2′-deoxyuridine cell proliferation assay

We performed an EdU assay to visualize the proliferating cells and used a Click-iT EdU Alexa Fluor 488 Imaging kit (Invitrogen Corp., Carlsbad, CA, United States) to detect cell proliferation as per the manufacturer’s instructions. We photographed EdU^+^ cells under a fluorescence microscope and counted them using ImageJ software (National Institutes of Health [NIH], Bethesda, MD, United States).

### Transwell invasion assay

We performed a transwell invasion assay according to previously described methods (Han et al., 2015). U87 and LN229 cell invasion was assessed using a Matrigel-coated filter over the lower compartment for 20 h. We counted the invading cells under a microscope (Olympus, Tokyo, Japan).

### Co-culture

Glioma cells and human brain microvascular endothelial cells (hBMECs) were co-cultured in Boyden chambers. Briefly, hBMECs were cultured in 6-well plates, while glioma cells were seeded in chambers.

### Tube formation assay

A pre-cooled 96-well plate was coated with 50 μl Matrigel (BD Biosciences, United States) per well and incubated at 37°C for 30 min. PBS was used to wash the tumor cells, and 0.25% trypsin was used for digestion. Cells were collected and counted using a hemocytometer after centrifugation. Then, the cells were resuspended with serum-free DMEM, and 2 × 10^4^ cells/well were inoculated on the surface of Matrigel. After 12 h, tube formation was photographed using a microscope (Olympus, Tokyo, Japan). ImageJ software was used to quantify and analyze tubule intersections.

### Statistical analysis

Statistical analyses and visualization were carried out in R. We performed time-dependent receiver operating characteristic (ROC) curve analysis to evaluate the predictive value of the constructed risk model using the R package survivalROC. The Wilcoxon test was used for comparisons between two groups, and the Kruskal–Wallis test was used for comparisons between multiple groups. A two-sided *p* < 0.05 was considered to be statistically significant.

## Results

### Consensus cluster analysis for angiogenesis-related gene expression profiles

**FIGURE 2:**
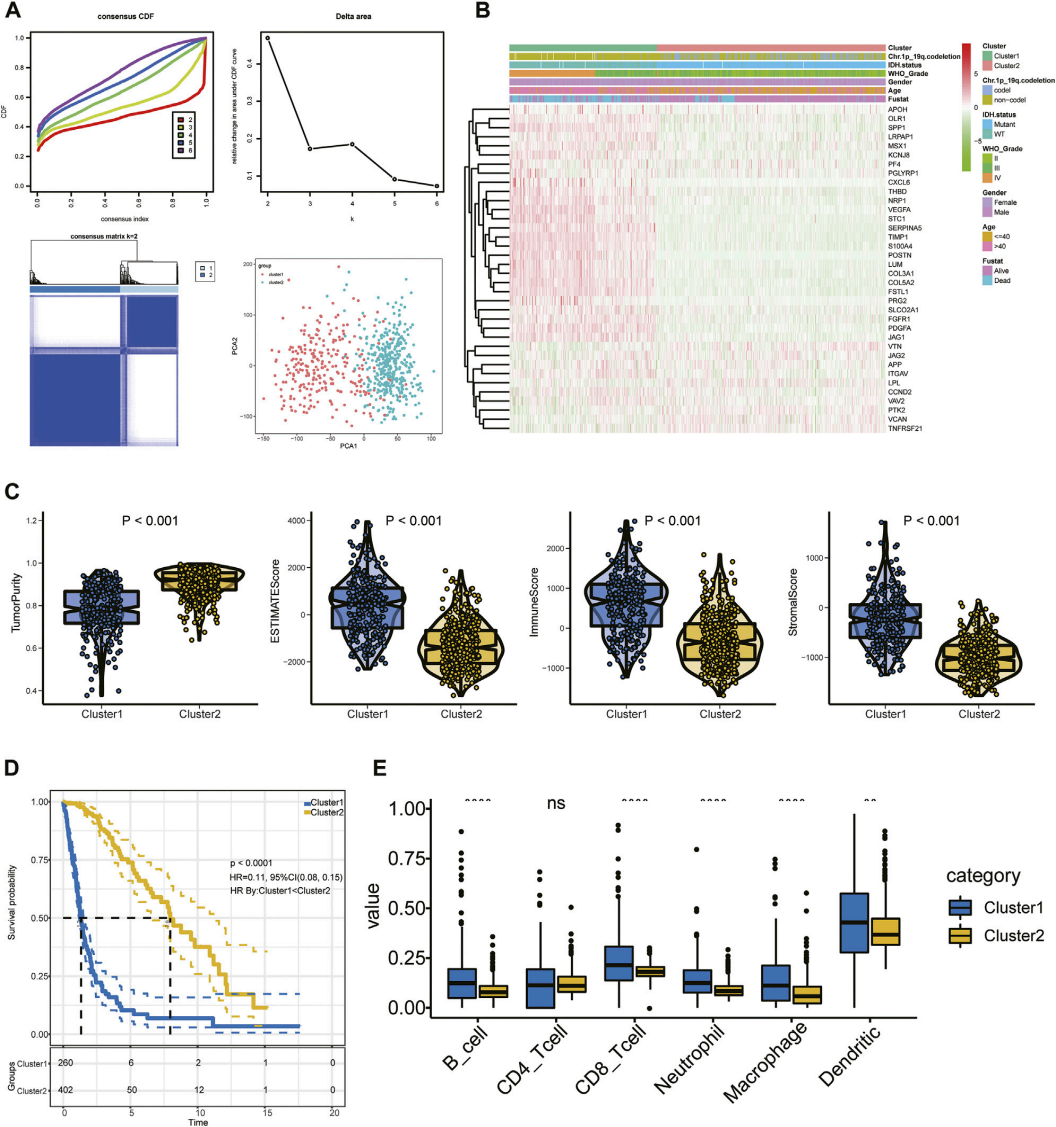
**(A)** Consensus clustering, CDF, and relative change in the CDF AUC with *k* = 2–6. **(B)** Heatmap of clinical information of the two clusters among 36 ARGs. **(C)** Tumor purity and ESTIMATE, stromal, and immune scores. **(D)** KM curve of glioma patients. **(E)** Content of six immune cells.

The set of ARGs we obtained from Gene Set Enrichment Analysis—Hallmark, Angiogenesis (GSEA) included 36 genes that are upregulated in tumorigenic angiogenesis (Subramanian et al., 2005; Ren et al., 2020). We performed consensus clustering in the glioma patient training cohort to analyze the prognostic implications of the ARGs (Figure 2A). The empirical cumulative-distribution function (CDF) plot revealed the lowest rangeability at 0.2–0.8, with *k* = 2 (Figure 2A); the delta area scores were the highest also at *k* = 2 (Figure 2A). In addition, the maximum consistency was found at *k* = 2 in the consensus matrix plot (Figure 2A; Supplementary Figure S1). Therefore, *k* = 2 was shown to have the best clustering stability. Cluster 1 (*n* = 260) and cluster 2 (*n* = 403) were generated from a total of 663 patients. We used principal component analysis (PCA) to display differences in gene expression levels between the two subgroups (Figure 2A). The heatmap shows the expression pattern of 36 ARGs in clusters 1 and 2 (Figure 2B). We found that immune score was significantly higher (*p* < 0.05), while tumor purity was significantly lower (*p* < 0.05) in cluster 1 than in cluster 2 (Figure 2C). Furthermore, a KM curve showed that the OS outcome of cluster 1 was worse than that of cluster 2 (Figure 2D). In addition, cluster 1 had significantly higher abundances of B cells, CD8^+^ T cells, neutrophils, macrophages, and DCs than cluster 2 (*p* < 0.05), while there was no between-cluster difference in CD4^+^ T cells (Figure 2E). These results indicated that the cluster assignment based on ARGs was closely related to prognosis and TIME in glioma.

### Establishment and validation of the risk signature based on angiogenesis-related gene expression

**FIGURE 3:**
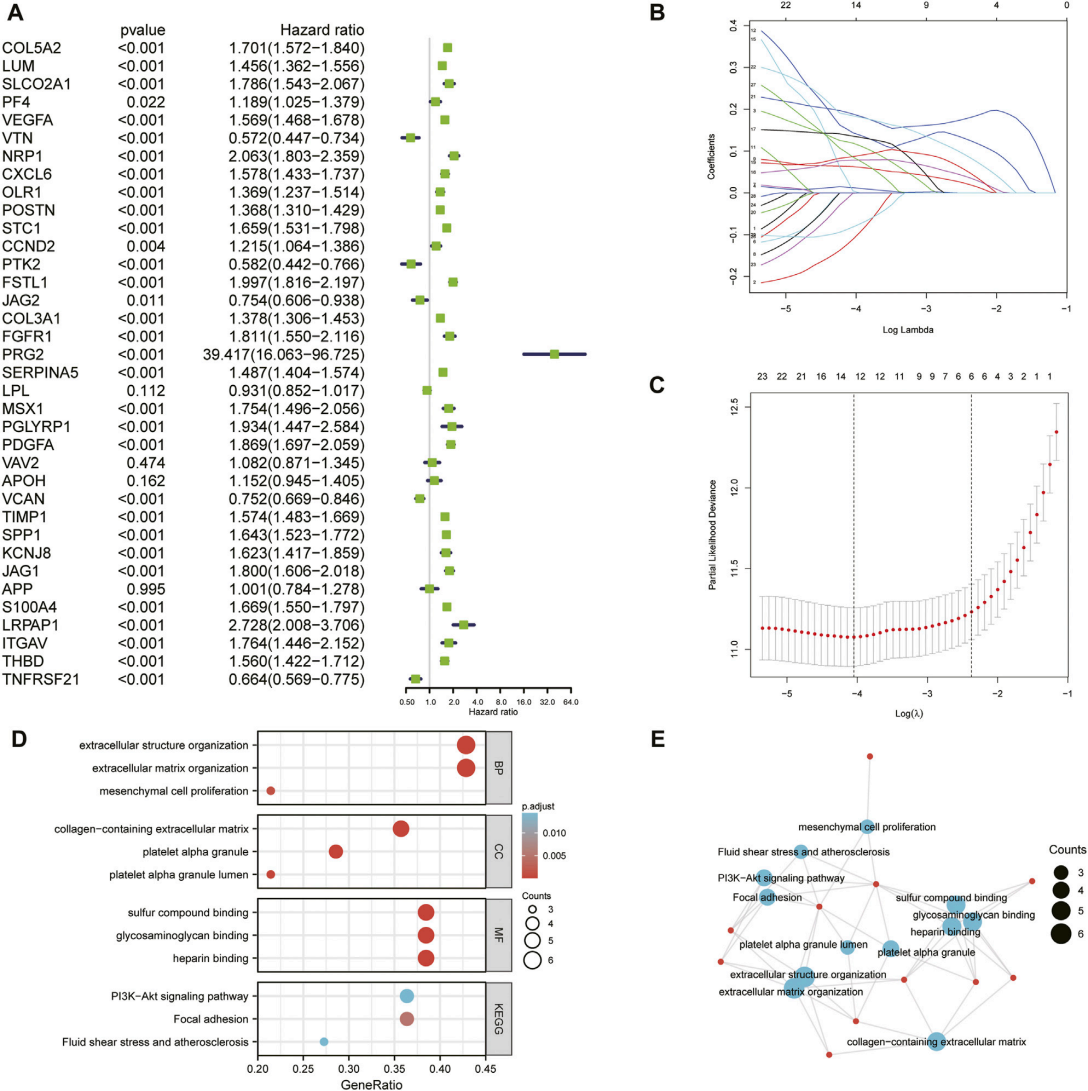
**(A)** Univariate Cox regression analysis of the 36 ARGs in the TCGA cohort. **(B)** LASSO coefficient profiles of the common genes. **(C)** Cross-validation for tuning parameter screening in the LASSO regression model. **(D)** GO and KEGG enrichment analysis across the 14 genes. **(E)** Functional-enrichment map of pathways of the 14 ARGs.

**FIGURE 4:**
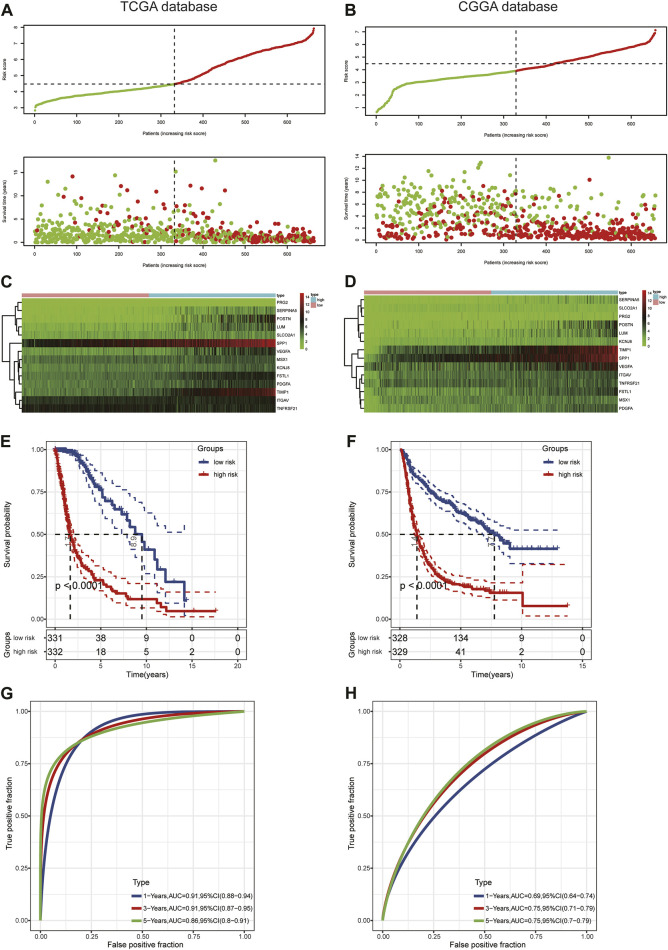
Prognostic value of the risk score in TCGA and CGGA. **(A,B)** Distribution of risk score and survival status. **(C,D)** Expression pattern of 14 ARGs in the high- and low-risk groups. **(E,F)** KM analysis of the risk model. **(G,H)** Time-dependent ROC curve analysis of the risk model.

First, we conducted univariate Cox regression analysis to screen out 29 OS-related ARGs (*p* < 0.001) in the TCGA cohort (Figure 3A). Subsequently, we selected these genes to conduct an additional least absolute shrinkage and selection operator (LASSO) Cox regression analysis (Figures 3B,C). The formula was as follows: risk score = (*LUM* × −0.11114) + (*SLCO2A1* × 0.11913) + (*VEGFA* × 0.01235) + (*POSTN* × 0.06287) + (*FSTL1* × 0.14389) + (*PRG2* × 0.00485) + (*SERPINA5* × 0.07829) + (*MSX1* × 0.13564) + (*PDGFA* × 0.08695) + (*TIMP1* × 0.1885) + (*SPP1* × 0.18423) + (*KCNJ8* × −0.00092) + (*ITGAV* × 0.08581) + (*TNFRSF21* × −0.0817). GO and KEGG enrichment analysis was performed by R package “clusterProfiler” (Yu et al., 2012). These genes were shown to be involved in extracellular structure organization and the PI3K-Akt signaling pathway (Figures 3D,E). Differential analysis was performed to detect 14 ARGs (Supplementary Figure S2). Patients in the training cohort (TCGA) were divided into high- and low-risk groups based on the median risk score. According to our findings, the number of patients who died increased as their risk score increased (Figures 4A,B). Differential expression levels of the 14 ARGs in the high- and low-risk groups are shown in heatmaps (Figures 4C,D). To evaluate the role of the 14-ARG signature in glioma, we drew KM curves for the high- and low-risk groups of the TCGA cohort (Figure 4E). These two subgroups significantly differed in OS (*p* < 0.0001). Thereafter, we used a time-dependent ROC curve to predict the efficacy of the risk signature. The area under the curve (AUC) of the prediction model was 0.91 over 1 year, 0.91 over 3 years, and 0.86 over 5 years in the TCGA training cohort (Figure 4G).

To assess the predictive value of the risk model, we used the risk score algorithm in the CGGA cohort. The results in the validation cohort revealed that glioma patients in the high-risk group had worse survival rates than those in the low-risk group (Figure 4F). The AUCs for 1-, 3-, and 5-year survival were 0.69, 0.75, and 0.75, respectively (Figure 4H). These findings suggested that the 14-ARG risk model could accurately predict the prognoses of patients with glioma.

### Association between angiogenesis-related gene risk signature and clinical information

**FIGURE 5:**
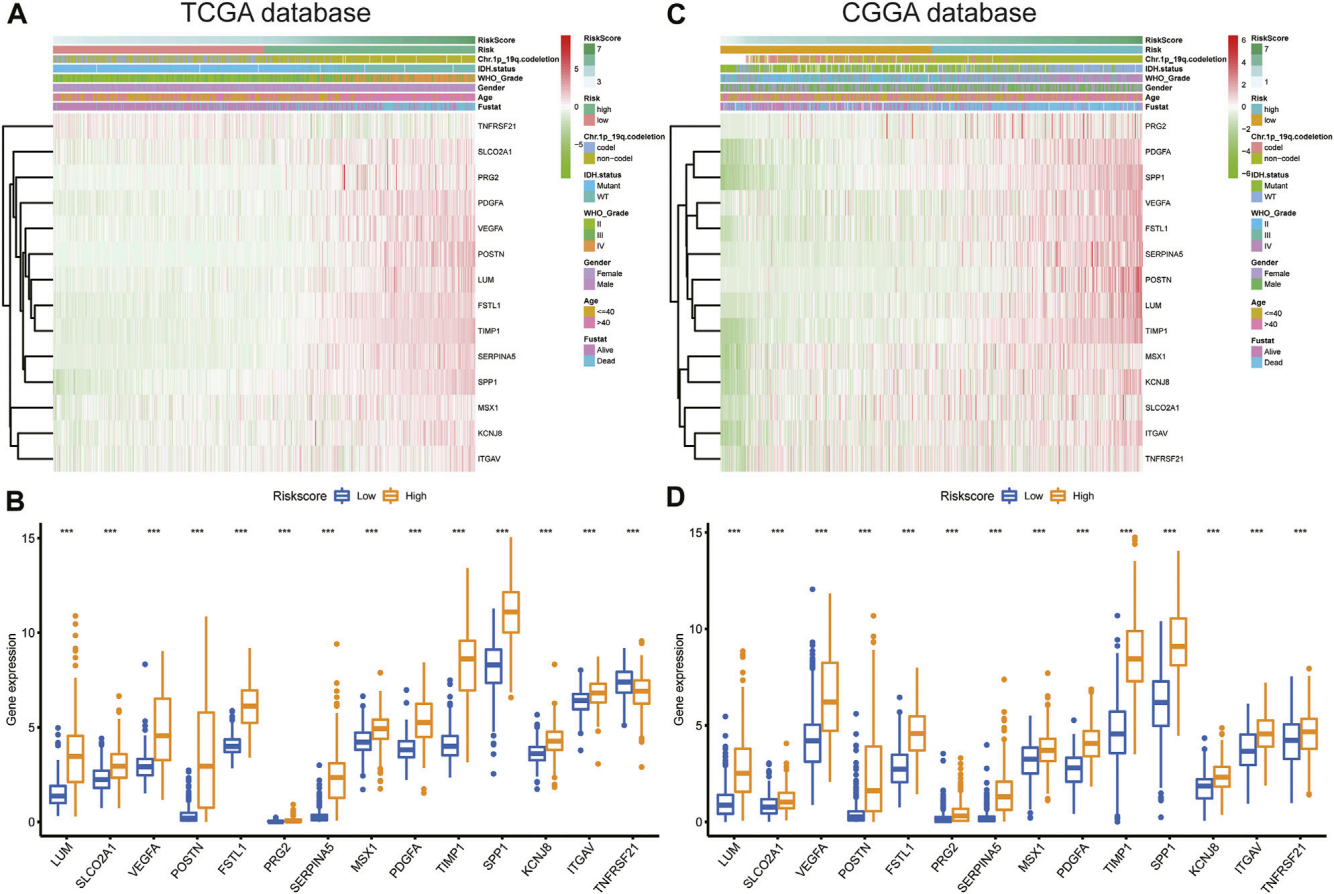
**(A,C)** Heatmap of the 14-ARG expression pattern in clinicopathologic characteristics and risk score in the TCGA and CGGA databases. **(B,D)** Expression differences in the 14 ARGs between the low- and high-risk groups in the TCGA and CGGA databases.

**FIGURE 6:**
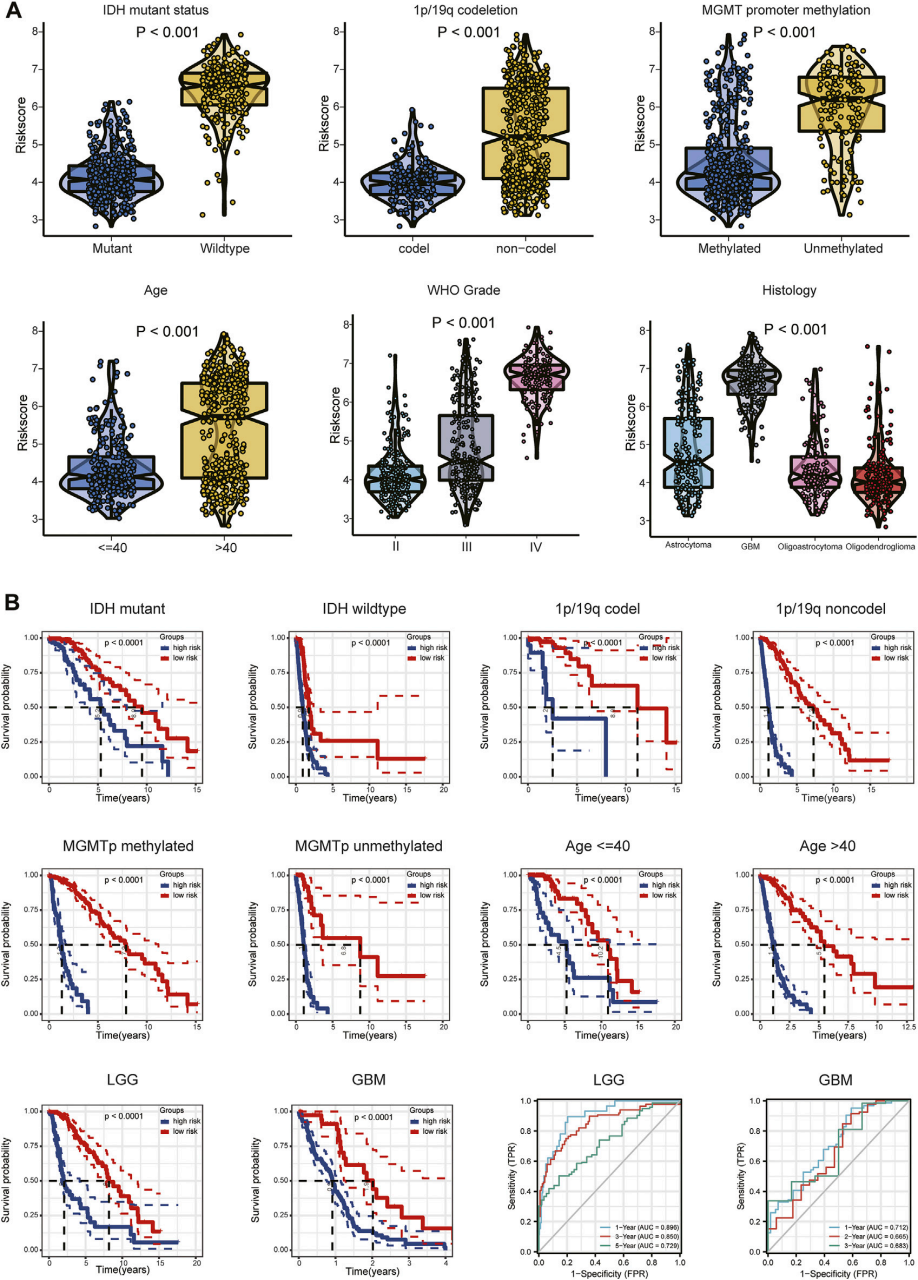
**(A)** Relationship between risk score and each clinicopathological characteristic (*IDH*-mutant status, 1p/19q codeletion, MGMT promoter methylation, age, WHO grade, and histology). **(B)** KM analyses of patients in the CGGA dataset stratified by *IDH*-mutant status, 1p/19q codeletion, MGMT promoter methylation, age, and WHO grade in the TCGA cohort. ROC curve analysis of the risk model in predicting 1-, 3- and 5-year OS in the TCGA–LGG cohort and 1-, 2- and 3-year OS in the TCGA–GBM cohort.

Expression of the 14 ARGs in low- and high-risk patients in the TCGA and CGGA datasets is depicted by heatmaps (Figures 5A,C). Other than those of *TNFRSF21*, expression of the 13 other ARGs increased significantly (*p* < 0.05) in the high-risk group (Figure 5B) of the TCGA cohort. All 14 ARGs were highly expressed in the high-risk group in the CGGA database (*p* < 0.05; Figure 5D). We also performed survival analysis of single ARGs in glioma patients (Supplementary Figures S3,S4). The results showed that for glioma patients in the TCGA cohort, all of the ARGs were prognostic-risk factors, except for *TNFRSF21*. Thereafter, we evaluated differences in risk score between different clinicopathological characteristics of glioma patients in the training and validation cohorts, including *IDH* mutation status, 1p/19q codeletion, MGMT promoter methylation, age, WHO grade, and histology. The results showed that in the TCGA dataset, the risk score was elevated in the *IDH* wild-type (WT), 1p/19q non-codeletion subtype, MGMT promoter unmethylated subtype, older patients, and high-grade glioma (*p* < 0.05); we validated these results in the CGGA dataset (Figure 6A; Supplementary Figure S5A). Next, we drew KM curves of the risk score signature stratified by *IDH*-mutant status, 1p/19q codeletion, MGMT promoter methylation, age, and WHO grade in the glioma patients of the training and validation cohorts. The KM curve suggested the predictive value of the ARG risk score signature in prognosis in the LGG and GBM subgroups (Figure 6B; Supplementary Figure S5B). The results demonstrated the power of the ARG risk score signature’s prognostic value in the glioma subgroups of the TCGA cohort (Figure 6B), and these results were consistent in the CGGA cohort (Supplementary Figure S5B).

Because different grades of glioma have different clinical features and prognoses, we performed subgroup analyses of LGG and GBM. The relationships between risk score and each clinical characteristic (*IDH*-mutant status, 1p/19q codeletion, MGMT promoter methylation, age) in the TCGA/CGGA-LGG and TCGA/CGGA-GBM subgroups are shown in Supplementary Figures S6A,S6D and in Supplementary Figures S7A,S7D, respectively. Tumor purity was significantly higher (*p* < 0.05) and ESTIMATE, immune, and stromal scores significantly lower (*p* < 0.05) in the low-risk group in the LGG and GBM subgroups (Supplementary Figures S6B,S6E, Supplementary Figures S7B,S7E). Expression differences of the 14 ARGs between the high- and low-risk groups of the LGG and GBM subgroups are shown in Supplementary Figures S6C,S6F and in Supplementary Figures S7C,S7F. The ROC curve showed the efficiency of the risk signature in these two subgroups. The AUC of the prediction model was 0.896 over 1 year, 0.850 over 3 years, and 0.729 over 5 years in the LGG subgroup and 0.712 over 1 year, 0.665 over 2 years, and 0.683 over 3 years in the GBM subgroup (Figure 6B; Supplementary Figure S5B). These results indicated the predictive stability of the 14-ARG risk score model’s prognostic value in both these subgroups.

**FIGURE 7:**
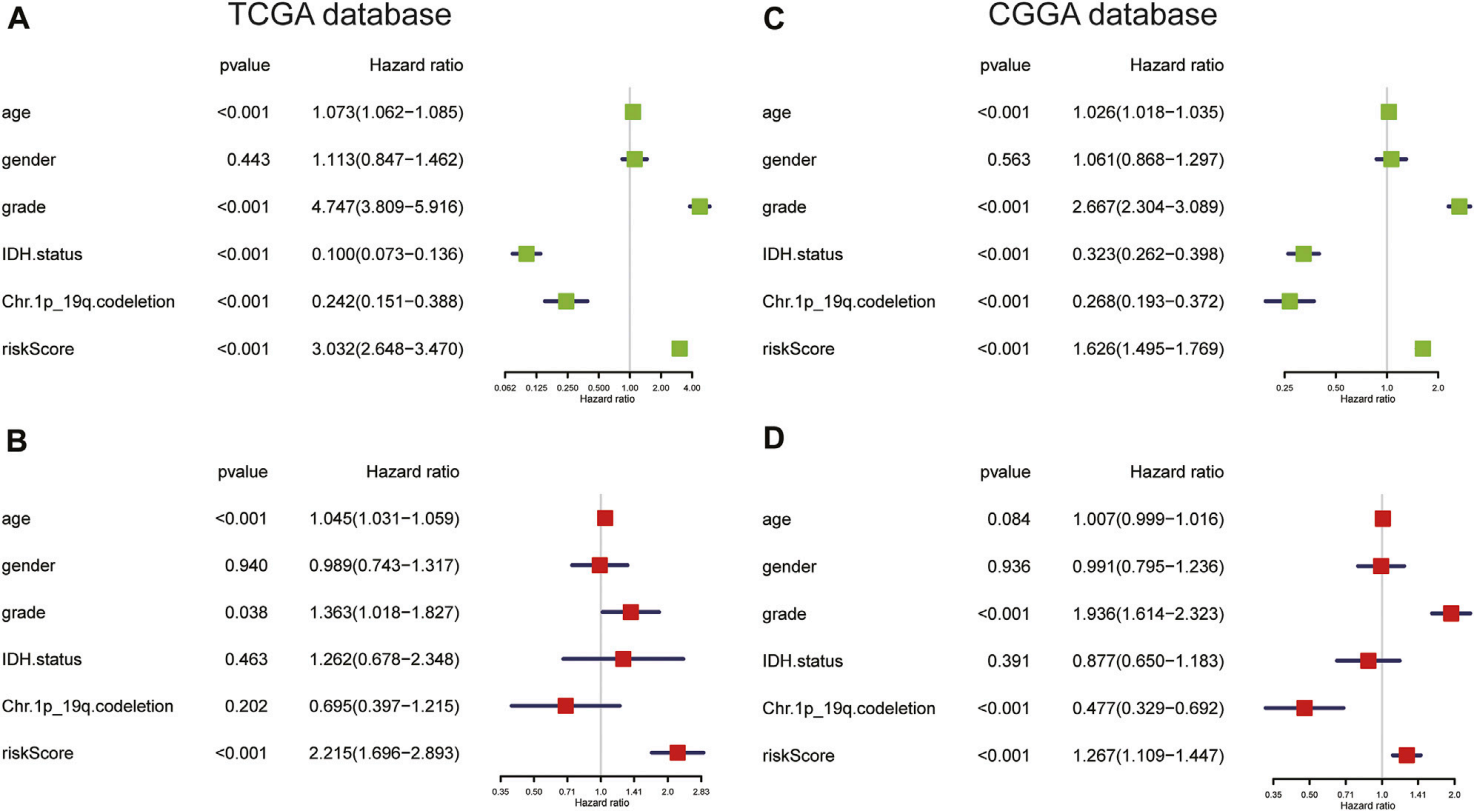
**(A,B)** Univariate and multivariate Cox regression analyses in the TCGA cohort. **(C,D)** Univariate and multivariate Cox regression analyses of clinicopathologic features in the CGGA cohort.

Next, we performed univariate and multivariate Cox regression analyses in the TCGA and CGGA cohorts to assess the independent prognostic value of the ARG risk signature. We observed that in univariate analysis, age, WHO grade, *IDH* status, chromosome 1p/19q status, and risk score were significantly correlated with prognosis in both the TCGA and CGGA cohorts (Figures 7A,C). However, multivariate analysis indicated that age, grade, and risk score were independent prognostic factors in the TCGA cohort (Figure 7B; *p* < 0.05). In the validation cohort (CGGA), we also found that risk score was an independent prognostic factor (Figure 7D; *p* < 0.05).

Furthermore, we compared the prognostic predictive abilities of 20 different risk signatures of gliomas in TCGA from published articles, including inflammatory response-related gene (IRRG) signature (Yan et al., 2022), DNA damage and repair-related gene (DDRRG) signature (Li et al., 2022c), CXCR members signature (He et al., 2022), pyroptosis-related gene signature (Zhang M. et al., 2021b; Chao et al., 2022; Yang et al., 2022; Zhang et al., 2022), ECM-related gene (ECMRG) signature (Li et al., 2022b), tripartite motif (TRIM) family gene signature (Xiao et al., 2022), antigen presentation machinery (APM) signature (Chen et al., 2022), natural killer cell-related gene (NKRG) signature (Li C. et al., 2022a), IL-4-related gene (IL4RG) signature (Qi et al., 2022), hypoxia-related gene (HRG) signature (Gao et al., 2021), S100 family-based signature (Hu et al., 2021), TIME signature (Zhang C. et al., 2021a), focal adhesion-related gene (FARG) signature (Li et al., 2021), m6A RNA methylation regulator signature (Cong et al., 2021), HDAC1-related signature (Fan et al., 2021), RNA-binding protein (RBP)-based signature (Chen et al., 2021a) and ferroptosis-related gene (FRG) signature (Chen et al., 2021b). The results of univariate and multivariate Cox analyses showed that our ARG signature had independent predictive ability (*p* < 0.001, Table 1).

**TABLE 1:** Univariate and multivariate Cox regression analyses of different risk signatures.

Characteristics	Univariate analysis		Multivariate analysis	
	Hazard ratio (95% CI)	*p* Value	Hazard ratio (95% CI)	*p* Value
Our ARG signature	3.032 (2.648–3.470)	**< 0.001**	3.019 (1.808–5.041)	**<0.001**
IRRG signature	13.574 (9.597–19.200)	**< 0.001**	0.785 (0.342–1.804)	0.569
DDRRG signature	6.885 (5.419–8.748)	**< 0.001**	2.899 (1.363–6.165)	**0.006**
CXCR member signature	1.251 (1.089–1.438)	**0.002**	0.971 (0.838–1.125)	0.698
PRG signature (Chao B *et al.*)	6.134 (4.858–7.745)	**< 0.001**	0.714 (0.387–1.321)	0.283
PRG signature (Yang Z *et al.*)	2.555 (2.257–2.892)	**< 0.001**	0.971 (0.698–1.350)	0.860
PRG signature (Zhang M *et al.*)	2.218 (2.013–2.444)	**< 0.001**	1.165 (0.825–1.645)	0.385
PRG signature (Zhang Y *et al.*)	2.751 (2.413–3.135)	**< 0.001**	1.177 (0.828–1.675)	0.364
ECMRG signature	5.518 (4.477–6.800)	**< 0.001**	0.455 (0.182–1.136)	0.092
TRIM family gene signature	23.500 (14.501–38.083)	**< 0.001**	0.904 (0.351–2.332)	0.835
APM signature	4.157 (3.333–5.185)	**<0.001**	0.578 (0.341–0.979)	**0.041**
NKRG signature	1195154.632 (130202.538–10970558.754)	**< 0.001**	5.857 (0.187–183.904)	0.315
IL4RG signature	266.447 (124.392–570.730)	**< 0.001**	0.738 (0.117–4.646)	0.746
HRG signature	2.974 (2.532–3.495)	**< 0.001**	0.923 (0.681–1.251)	0.606
S100 family-based signature	2.833 (2.475–3.244)	**< 0.001**	0.784 (0.524–1.172)	0.235
TIME signature	5.365 (4.355–6.607)	**< 0.001**	1.365 (0.704–2.646)	0.357
FARG signature	2.974 (2.502–3.535)	**< 0.001**	0.689 (0.510–0.931)	**0.015**
m6A RNA methylation regulators signature	3.852 (3.236–4.586)	**< 0.001**	0.845 (0.562–1.269)	0.416
HDAC1-related signature	3.605 (3.033–4.284)	**< 0.001**	1.158 (0.796–1.685)	0.444
RBP-based signature	3.130 (2.673–3.664)	**< 0.001**	1.081 (0.808–1.445)	0.602
FRG signature	2.786 (2.456–3.159)	**< 0.001**	1.569 (1.053–2.338)	**0.027**

The bold values are *p* < 0.05.

Based on the abovementioned comprehensive analyses, we considered the effect of risk score on prognosis to be accurate and stable.

### Angiogenesis-related gene risk signature and the tumor immune microenvironment

**FIGURE 8:**
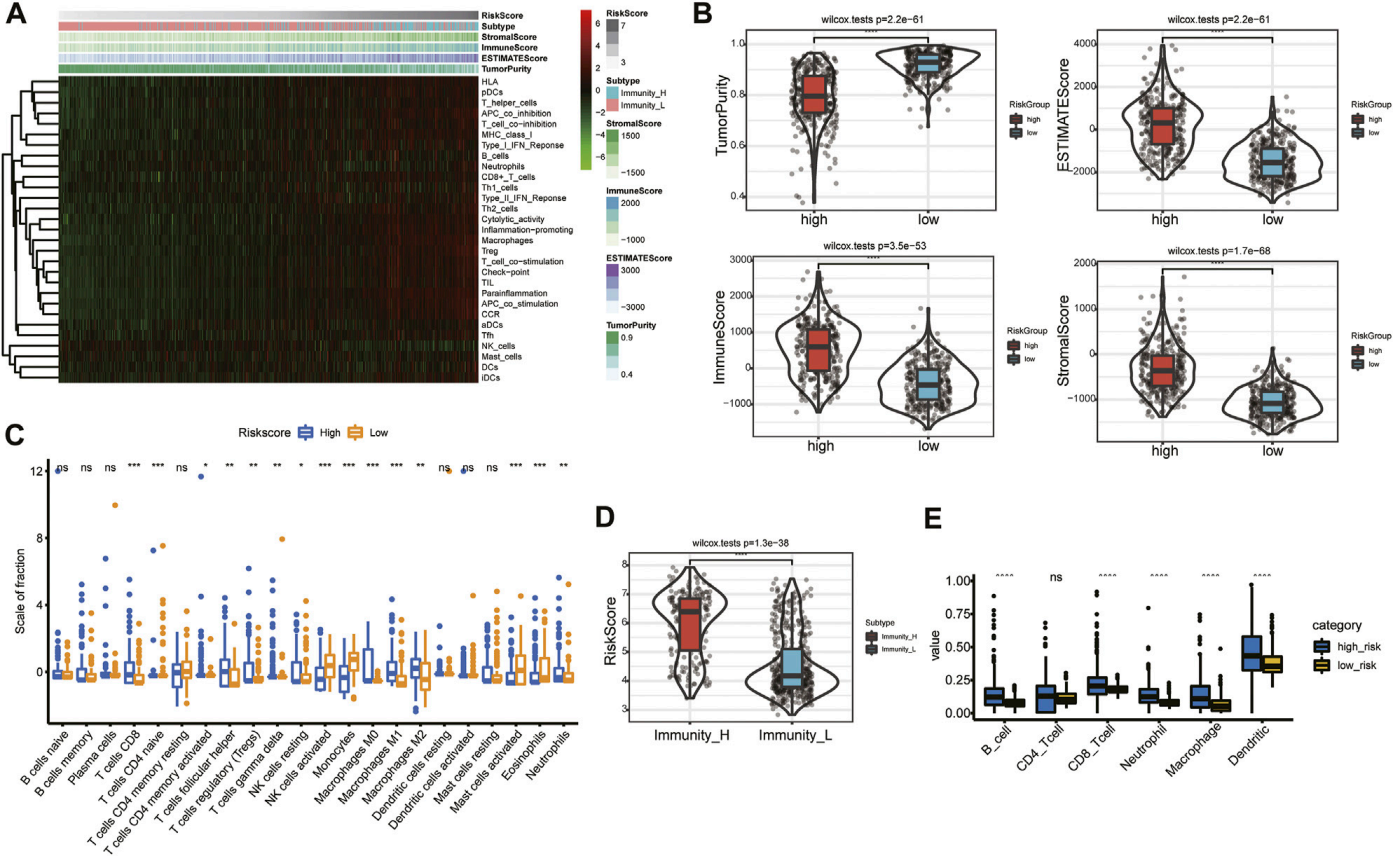
Relationship between risk signature and TIME in the TCGA database. **(A)** Heatmap of risk score and the two immunity subtypes based on ssGSEA. **(B)** Comparison of tumor purity and of ESTIMATE, immune, and stromal scores in the high- and low-risk groups. **(C)** Association between immune cells and the risk signature. **(D)** Comparison of risk score between the immunity-high and immunity-low subtypes. **(E)** Abundances of six immune cells in the high- and low-risk groups.

The heatmap of immune responses based on the ESTIMATE algorithms and single-sample GSEA (ssGSEA) is depicted in Figure 8A. Tumor purity was substantially lower (*p* < 0.05) in the high-risk group, but ESTIMATE, immune and stromal scores were significantly higher (Figure 8B). We calculated the proportions of 22 types of immune cells in each glioma sample based on the CIBERSORT algorithm. Next, we compared differences in proportions of immune cells between the high- and low-risk groups in the TCGA database. Abundances of CD8^+^ T cells, follicular helper T (T_f_h) cells, regulatory T cells (Tregs), gamma delta (γδ) T cells, resting natural-killer (NK) cells, M0, M1, and M2 macrophages, and neutrophils were significantly more enriched in the high-risk than in the low-risk group (Figure 8C). Additionally, we identified two immune subtypes based on immune-genomic profiling of 29 immune signatures in ssGSEA. We found a significantly higher risk score in the immunity-high subtype than the immunity-low subtype (Figure 8D). We also compared six immune cell types *via* the TIMER algorithm, and results showed that abundances of B cells, CD8^+^ T cells, neutrophils, macrophages, and DCs were significantly higher in the high-risk group (Figure 8E). We obtained similar TIME infiltration results in the validation cohort (Supplementary Figure S8), indicating greater infiltration of CD8^+^ T cells, T_f_h cells, Tregs, and M0 macrophages in the high-risk group (Supplementary Figure S8C), and risk score remained higher in the immunity-high subtype (Supplementary Figure S8D). These results demonstrated that the ARG risk signature was closely associated with infiltration of immune cells.

### Angiogenesis-related gene risk signature and mutational profile

**FIGURE 9:**
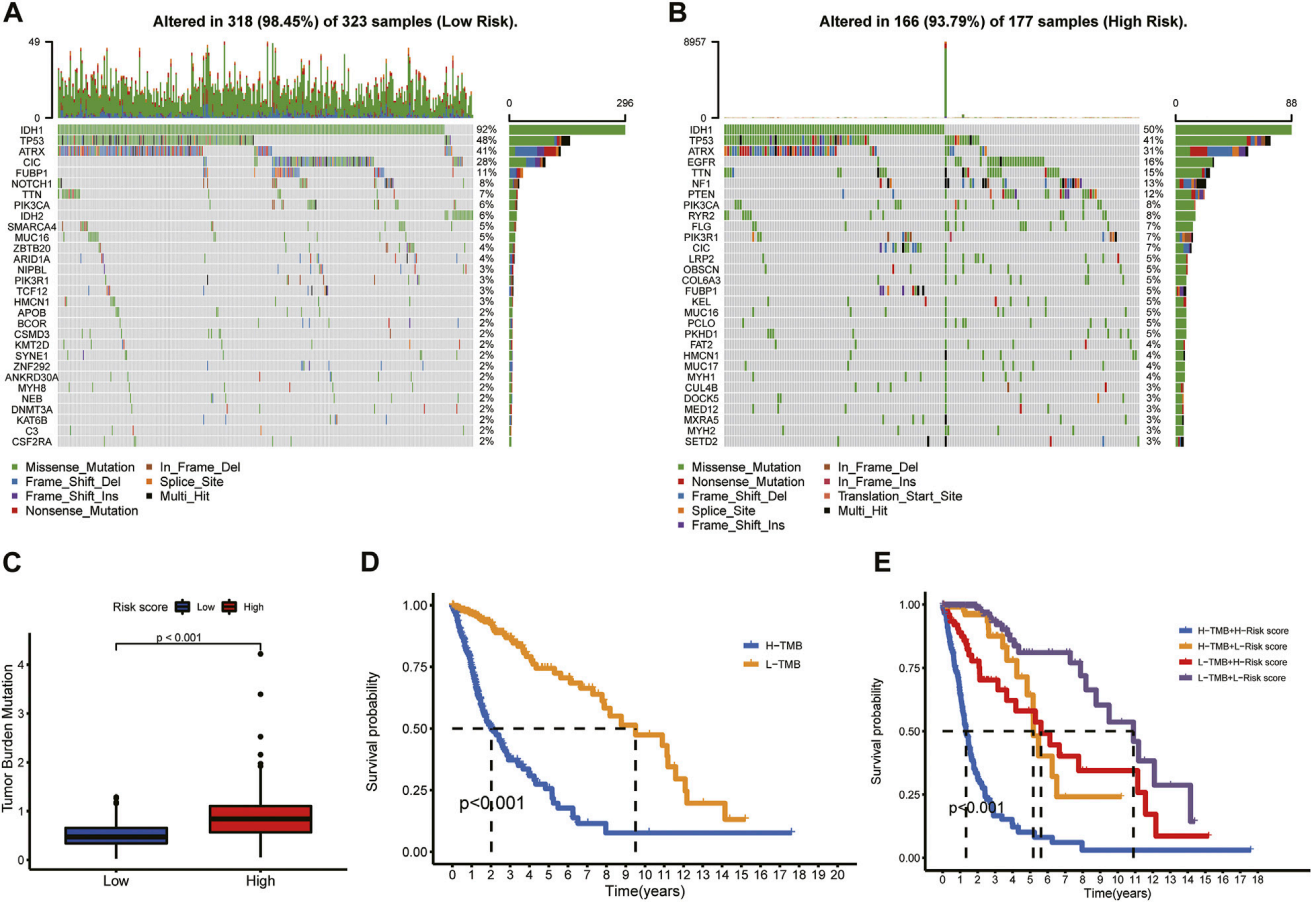
Mutational profile and TMB in the low- and high-risk groups. **(A)** Mutational profile in the low-risk group. **(B)** Mutational profile in the high-risk group. **(C)** Difference in TMB between low- and high-risk groups. **(D)** KM analysis of the high- and low-TMB groups. **(E)** Survival curve of the TMB combined risk score.

The mutational landscapes between the low- and high-risk groups of each glioma patient in TCGA were analyzed and are displayed as a waterfall plot (Figures 9A,B). Compared with the low-risk group, TMB was significantly high (*p* < 0.001) in the high-risk group (Figure 9C). A log rank test and the KM curve showed that the high-TMB group had worse survival outcomes than the low-TMB group (*p* < 0.001; Figure 9D). We also drew the survival curve of the TMB combined risk score (Figure 9E); the results showed that the high-TMB plus high-risk score group had a worse survival outcome (*p* < 0.001).

### Angiogenesis-related gene risk signature and immunotherapy

**FIGURE 10:**
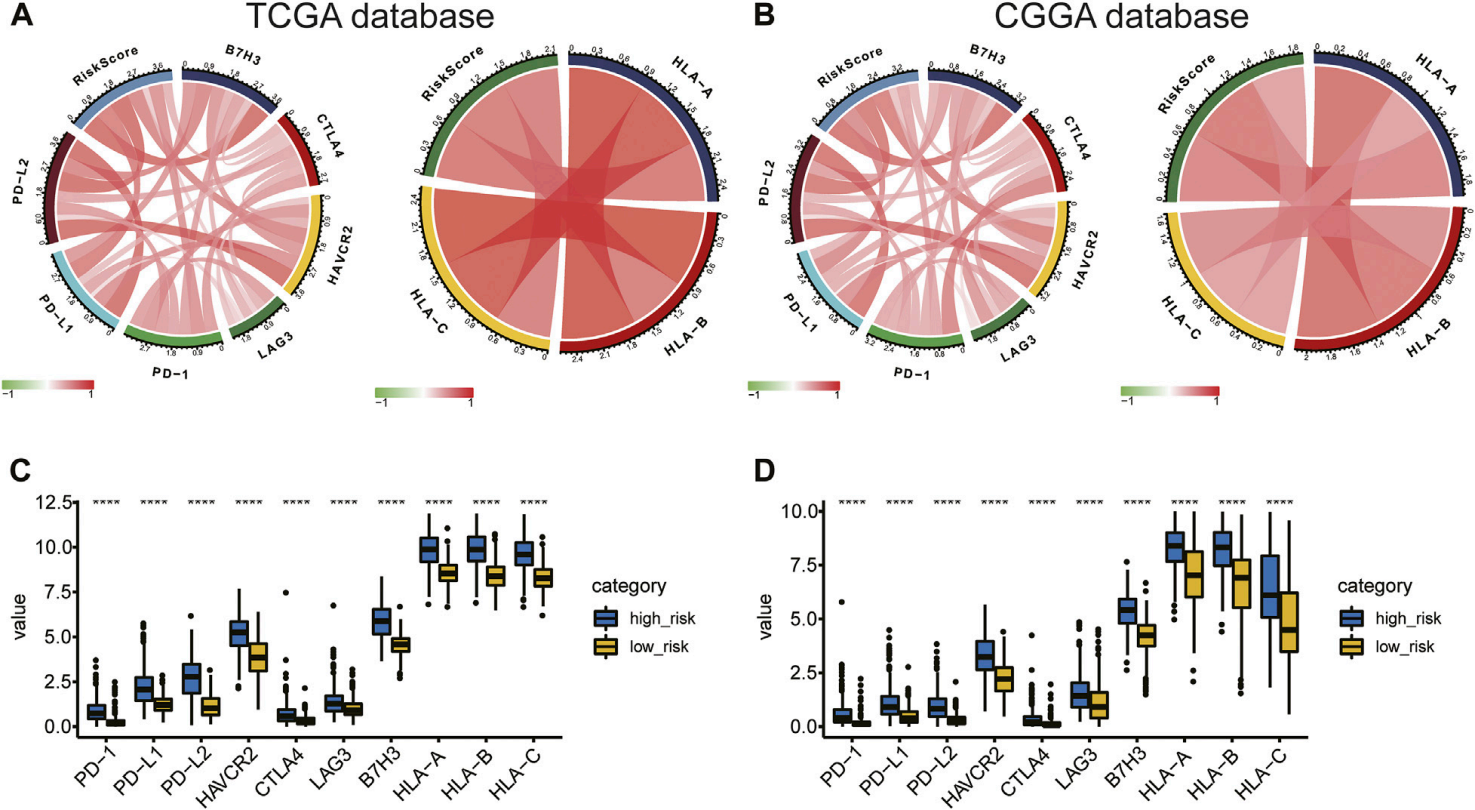
**(A,B)** Correlation of risk score to immune checkpoints and *HLA* complex expression levels. **(C,D)** Difference in expression of immune checkpoints and the *HLA* complex between the high- and low-risk groups.

The association between risk score and immunotherapeutic effect was also explored. We found that risk scores were positively correlated with expression of crucial immune checkpoints (*B7H3*, *PD-L1*, *PD-L2*, *HAVCR2*, *LAG-3*, *PD-1*, *CTLA4,* and the inflammatory factors *HLA-A*, *HLA-B*, and *HLA-C*) in the TCGA and CGGA databases (Figures 10A,B). Furthermore, we evaluated immune checkpoint and *HLA* complex expression levels. The high-risk group of the training and validation cohorts had considerably greater expressions of both. (*p* < 0.05; Figures 10C,D). Collectively, the results suggested that risk stratification could help predict the effect of immunotherapy in gliomas.

### Construction and validation of the prognostic-nomogram model

**FIGURE 11:**
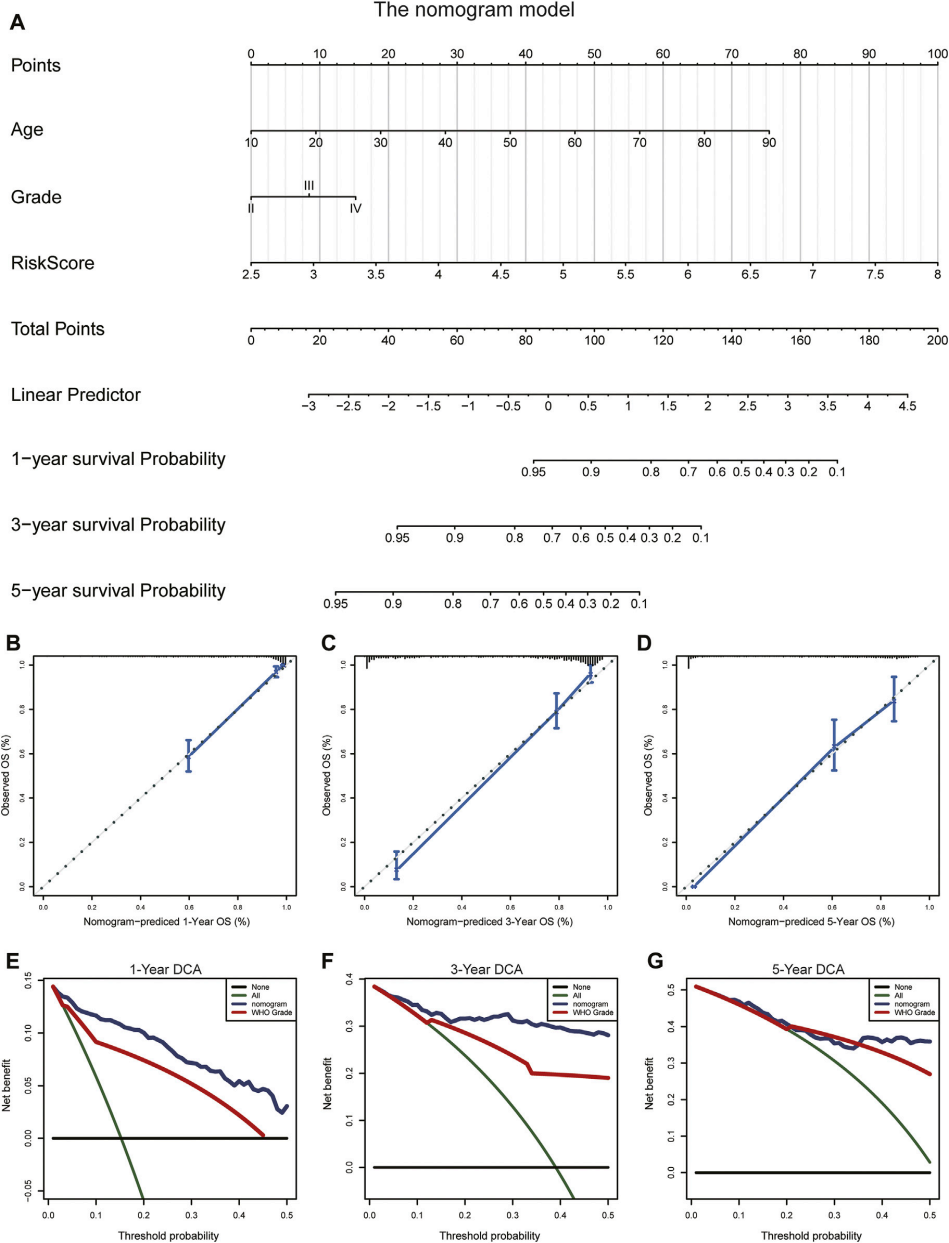
Construction and validation of the nomogram to predict OS in glioma patients. **(A)** The nomogram was established using age, WHO grade, and the ARG risk signature in the TCGA cohort. **(B–D)** Calibration curve of the nomogram for predicting the probability of OS at 1, 3, and 5 years in the TCGA cohort. **(E–G)** DCA of the OS-related nomogram at 1, 3, and 5 years in the TCGA cohort.

To evaluate the prognostic significance of the ARG signature in glioma patients, we established a nomogram model based on age, WHO grade, and risk score (Figure 11A; Supplementary Figure S9A) using our multivariate-analysis results. The C-index of the nomogram model was generated to assess discriminating abilities, and it performed well (TCGA training cohort, 0.875; CGGA validation cohort, 0.735). In the TCGA and CGGA cohorts, the calibration curves revealed a favorable consistency between expected and observed survival rates (Figures 11B–D; Supplementary Figures S9B–D). In addition, we used DCA to examine the suitability of the nomogram in clinical settings. The model exhibited an excellent net benefit (Figures 11E–G; Supplementary Figures S9E–G). Taken together, the results described above suggested that the nomogram model had good reliability in predicting OS in glioma patients.

### Knockdown of *SPP1* significantly inhibited cell proliferation, invasion, and angiogenesis

**FIGURE 12:**
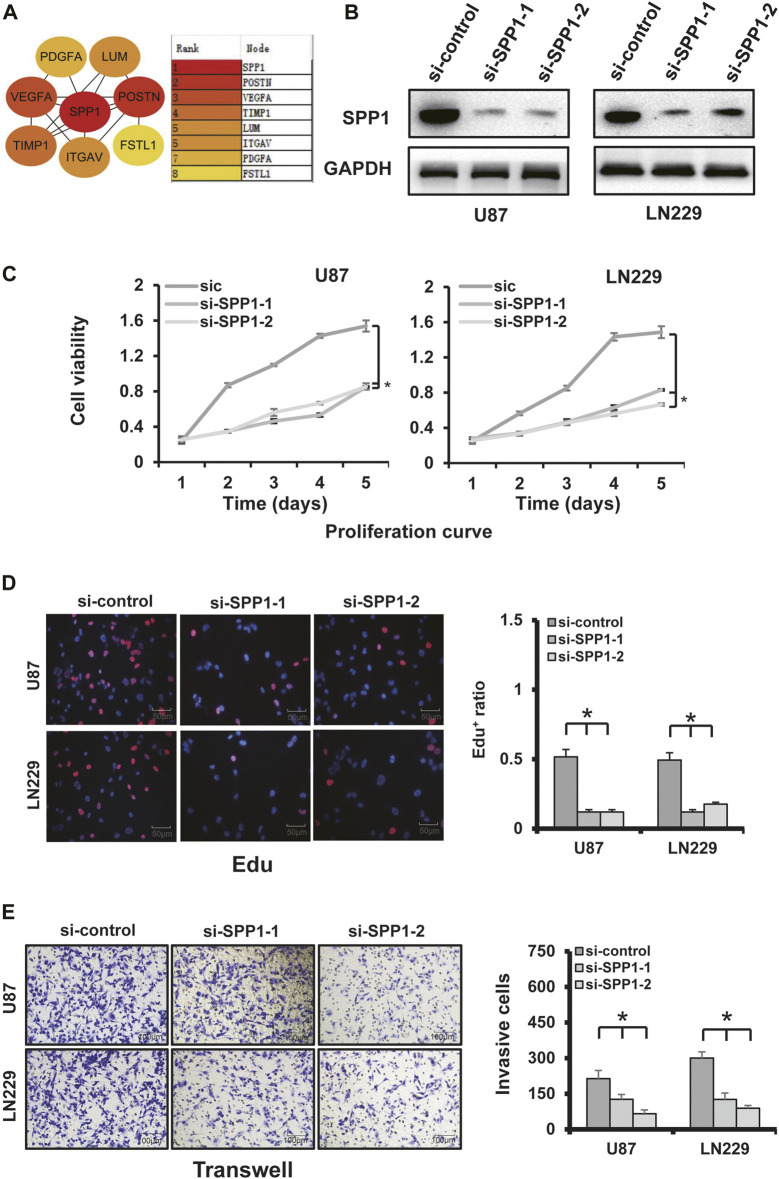
*SPP1* experiments. **(A)** PPI analysis and the MCC method of cytoHubba showed that *SPP1* had the highest hub node score. **(B)** *SPP1* knockdown using two independent *SPP1* siRNAs (si-*SPP1*-1, si-*SPP1*-2) in U87 and LN229 cells was evidenced by WB analysis. *GAPDH* was using as loading control. **(C)** Cellular-viability assays demonstrated that silencing *SPP1* inhibited the growth of U87 and LN229 cells. **(D)** Representative images of cellular-proliferation assays using EdU staining (left) and quantification of EdU^+^ cells (right). Nuclei were counterstained with Hoechst 33,342 (scale bar: 50 μm). **(E)** Matrigel assay demonstrated that knockdown of *SPP1* inhibited U87 and LN229 invasion (scale bar: 100 μm).

**FIGURE 13:**
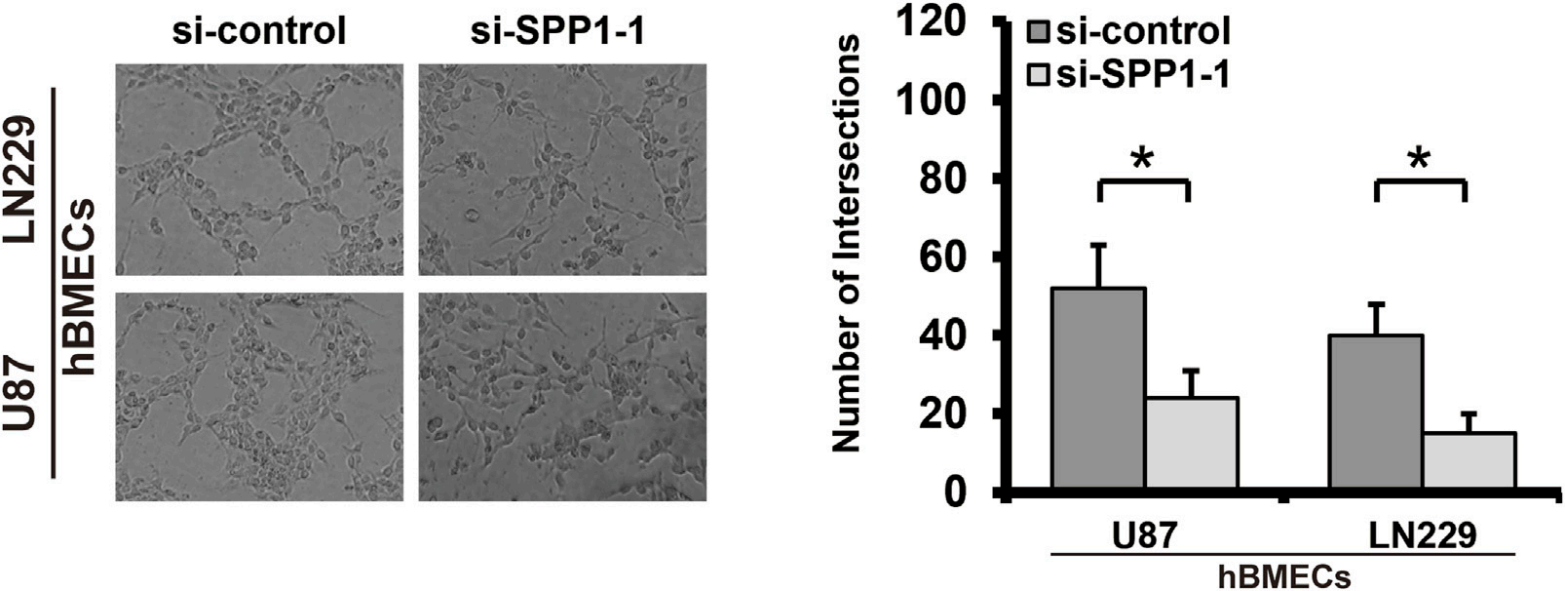
Tube formation assay. Knockdown of *SPP1* inhibited tumor angiogenesis *in vitro.* All experiments were performed in triplicate.

*SPP1* was overexpressed in the high-risk group of glioma patients and was correlated with poor prognosis. The results of PPI analysis and the MCC method of cytoHubba suggested *SPP1* may be the hub gene (Figure 12A). In the U87 and LN229 glioma cell lines, we determined the role of *SPP1* using *in vitro* experiments. SiRNA was used to reduce expression of *SPP1* in both U87 and LN229 cells; *SPP1* protein expression levels are shown in Figure 12B. We used a cellular-viability assay to analyze the effects of *SPP1* on the proliferation of U87 and LN229 cells. The results, which were presented as the mean ± standard deviation (SD) of three independent experiments, suggested that *SPP1* knockdown significantly reduced the viability of glioma cells (Figure 12C; *p* < 0.05). Meanwhile, the results of EdU assay suggested that *SPP1* inhibited the proliferation capacity of the glioma cell lines (Figure 12D). Transwell experiments suggested that knockdown of *SPP1* could also inhibit migration and invasion of U87 and LN229 cells Figure 12E). hBMECs co-cultured with si-*SPP1* glioma cells showed attenuated network formation when compared with controls (Figure 13), which suggested knockdown of *SPP1* inhibited angiogenesis.

## Discussion

Despite advances in surgical and medical treatment, glioma remains a fatal disease. Numerous studies indicate that aberrant angiogenesis is involved in the processes of tumorigenesis, development, invasion, and poor prognosis in glioma (Tan et al., 2018). To date, there are still few studies on ARG in glioma (Biterge-Sut, 2020; Wang et al., 2022). Two major aspects of glioma biological processes that contribute to treatment resistance are abnormal formation of new blood vessels via angiogenesis and invasion of glioma cells along white-matter tracts (Carmeliet, 2005; Onishi et al., 2011). Although using immunohistochemistry (IHC) to analyze the expression level of a single angiogenesis gene is convenient (Tan et al., 2018; Peng et al., 2021), multi-gene signature analysis can reveal the complex interactions among various factors that affect angiogenesis in the pathophysiology of gliomas. Therefore, application of multi-gene methods might help researchers better describe the characteristics of tumor biology, thereby guiding clinical decision-making for accurate cancer diagnosis and treatment. The effectiveness of single-ARG targeted treatment is still limited (Onishi et al., 2011), suggesting that angiogenesis in glioma likely results from multiple genes and factors and that exploration of multi-gene signatures might provide guiding significance for multi-target combined therapy.

In this study, we performed consensus clustering based on the ARG expression level to create two clusters. KM analysis showed that glioma patients in cluster 1 had unfavorable clinical outcomes. Moreover, immune cell infiltration in cluster 1 was greater than that in cluster 2. These results indicated that high immune scores and high infiltration of immune cells were correlated with poor prognosis, which was consistent with that in previous studies (Deng et al., 2020; Tian et al., 2020; Xu et al., 2021). Next, we identified 14 ARGs of significance and applied them to build a risk model by combining LASSO and Cox regression analyses. The risk score showed a favorable predictive value for the survival rate of glioma patients in the training and validation cohorts. Moreover, the risk score was found to be an independent predictor of glioma prognosis in multivariate Cox regression analyses. Furthermore, we established and validated a nomogram model to predict OS in glioma. The calibration curve revealed high concordance between predicted and actual OS rates, indicating good prediction performance of the nomogram model.

The biological functions of 14 ARGs have been moderately studied in various cancers, but not as much in gliomas. Crocker et al., (2011) found that *TIMP-1* serum level is positively correlated with *TIMP-1* expression in tumor tissue and inversely correlated with survival time of glioma patients. *VEGFA* is a critical target of anti-angiogenic treatment for a variety of malignant tumors, including gliomas, since it is a fundamental mediator of tumor angiogenesis (Tamura et al., 2019). In addition to angiogenesis, *VEGFA* can inhibit the maturation of DCs to inhibit tumor immune response and induce immunosuppressive cells (Lindau et al., 2013). Previous research studies have shown that elevated *VEGFA* expression levels are related to poor prognosis in many tumors, including gliomas (Hicklin and Ellis, 2005). Reddy et al., (2008) found that overexpression of *FSTL1* is a biomarker of poor prognosis in GBM patients, and Jin et al., (2017) demonstrated that this gene is a critical modulator that promotes cell proliferation and cell cycle progression. Overexpression of *SPP1* is associated with poor OS in patients with glioma (Chen et al., 2019). The results of our functional experiments showed that *SPP1* knockout could inhibit the proliferation, invasion, and angiogenesis of glioma cell lines U87 and LN229. Therefore, we believe that *SPP1* might affect the prognosis of glioma by helping regulate angiogenesis and cell proliferation. The abovementioned evidence indicated that the 14 ARGs might play important roles in angiogenesis, invasiveness, and the TIME of gliomas. This also suggested that the ARG risk signature could help support clinical decision-making in glioma patients.

Previous studies have shown that immune infiltration plays an important role in determining therapeutic effect and prognosis in glioma patients (Gentles et al., 2015; Pereira et al., 2018; Kruger et al., 2019; Xu et al., 2020b). Tumor angiogenesis facilitated by hypoxia in the TIME leads to an antitumor immune response (Abou Khouzam et al., 2020). Macrophages are abundant cell components in the glioma microenvironment, which can promote proliferation, invasion, and migration of glioma (Uneda et al., 2021). Researchers have found that a high level of infiltrating CD8^+^ T cells is correlated with poor prognosis in glioma (Zhai et al., 2017; Weenink et al., 2019; Guo et al., 2020). Therefore, we further explored the relationship between immune cell infiltration and risk stratification. Data from the ESTIMATE algorithm showed that ARG risk stratification was negatively correlated with tumor purity and positively correlated with immune and stromal scores, which suggested higher infiltration levels of immune and stromal cells in the TME of the high-risk group. Numerous studies have shown that TAMs might promote the proliferation and progression of gliomas by enhancing immunosuppression, migration, invasion, and angiogenesis (Li and Graeber, 2012; Coniglio and Segall, 2013; Kennedy et al., 2013; Zhang Y. et al., 2021c). In our study, we found that the high-risk group had a higher infiltration of immunosuppressive cells such as M2 macrophages and Tregs, which create an immunosuppressive microenvironment and inhibit NK cell activation. The abundance of activated NK cells in the high-risk group was lower than that in the low-risk group. In general, we speculate that the poor prognosis of glioma patients in the high-risk group might be related to the tumor immunosuppressive microenvironment.

Multiple studies have reported that glioma acquires aggressive characteristics depending on a series of genome alterations (Kim et al., 2015; Yin et al., 2020). TMB has become a novel potential biomarker for predicting the efficacy of immune checkpoint therapy in many cancers (Braun et al., 2016; Chan et al., 2019). We explored the mutational profiles and TMBs of the high- and low-risk groups to investigate the predictive value of the risk model. We found that TMB increased significantly in the high-risk group and that patients with high TMB had poor prognoses. Consistent with our findings, Yin et al., (2020) found that TMB is negatively correlated with OS in glioma patients. Previous studies have suggested that immune checkpoints and the *HLA* complex have been implicated in the treatment response and prognosis of glioma (Luoto et al., 2018; Cloughesy et al., 2019; Feng et al., 2019). Kim et al., (2020) found that *HAVCR2* (*TIM-3*) plays specific intracellular and intercellular immunoregulatory roles in the TME of gliomas. Studies have shown that the *HLA* level is positively related with development of gliomas (Machulla et al., 2001). In this study, risk score was positively correlated with expression of immune checkpoint molecules and *HLA* complex. These findings demonstrated the 14-ARG risk model’s accuracy in the prediction of the TIME of glioma, which therapeutic targets based on this signature might alter. The ARG expression signature could be used to predict clinical prognosis and efficacy of immunotherapy in glioma patients, and it might itself constitute a potential therapeutic target.

## Conclusion

In summary, the study analyzed the expression pattern and predictive value of ARGs in gliomas. Furthermore, we used a risk model based on the expression of ARGs to predict survival, and the risk score was correlated with the TIME in gliomas. The risk score can be used as an independent prognostic indicator. However, further studies using prospective, large-scale, multicenter clinical cohorts are needed to validate the risk model.

## Data Availability

The original contributions presented in the study are included in the article/Supplementary Material; further inquiries can be directed to the corresponding authors.
