# Supplementary figures and images for: Construction and validation of an angiogenesis-related gene expression signature associated with clinical outcome and tumor immune microenvironment in glioma
Source: Front Genet. 2022 Aug 11;13:934683. doi: 10.3389/fgene.2022.934683 (PMC9403517; doi:10.3389/fgene.2022.934683)

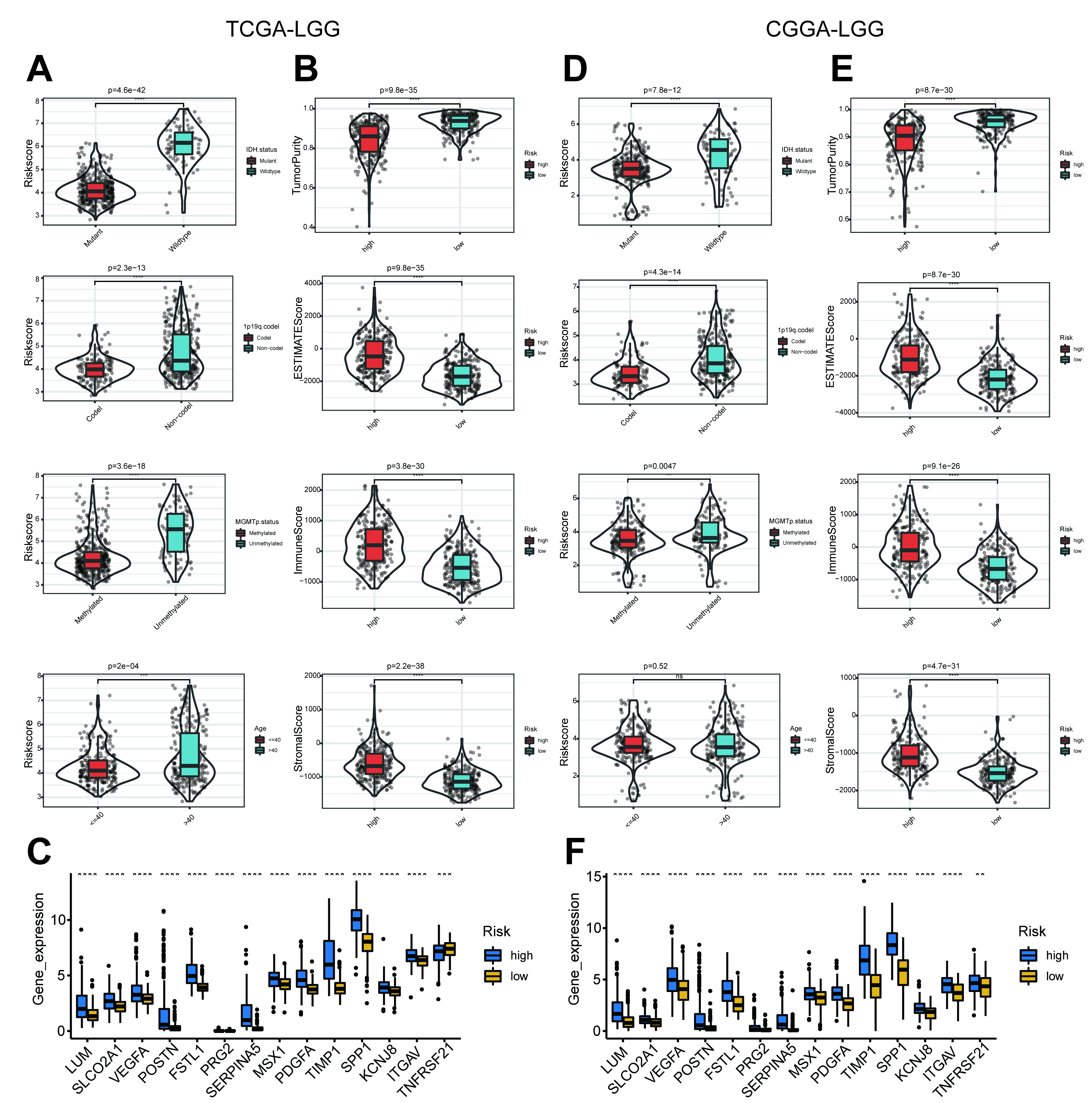

Supplement: Supplementary file 1 [file Image6.TIF]

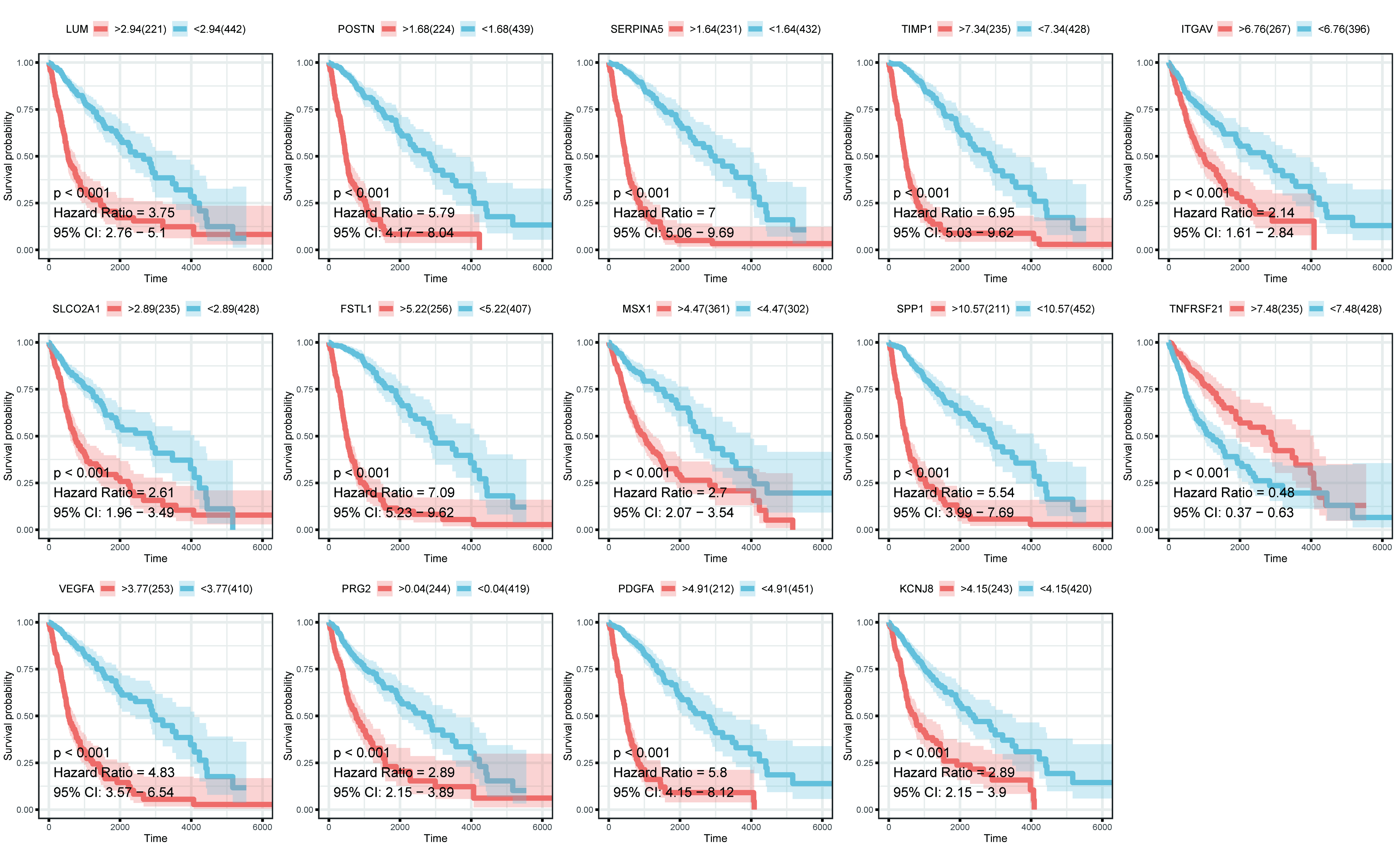

Supplement: Supplementary file 2 [file Image3.TIF]

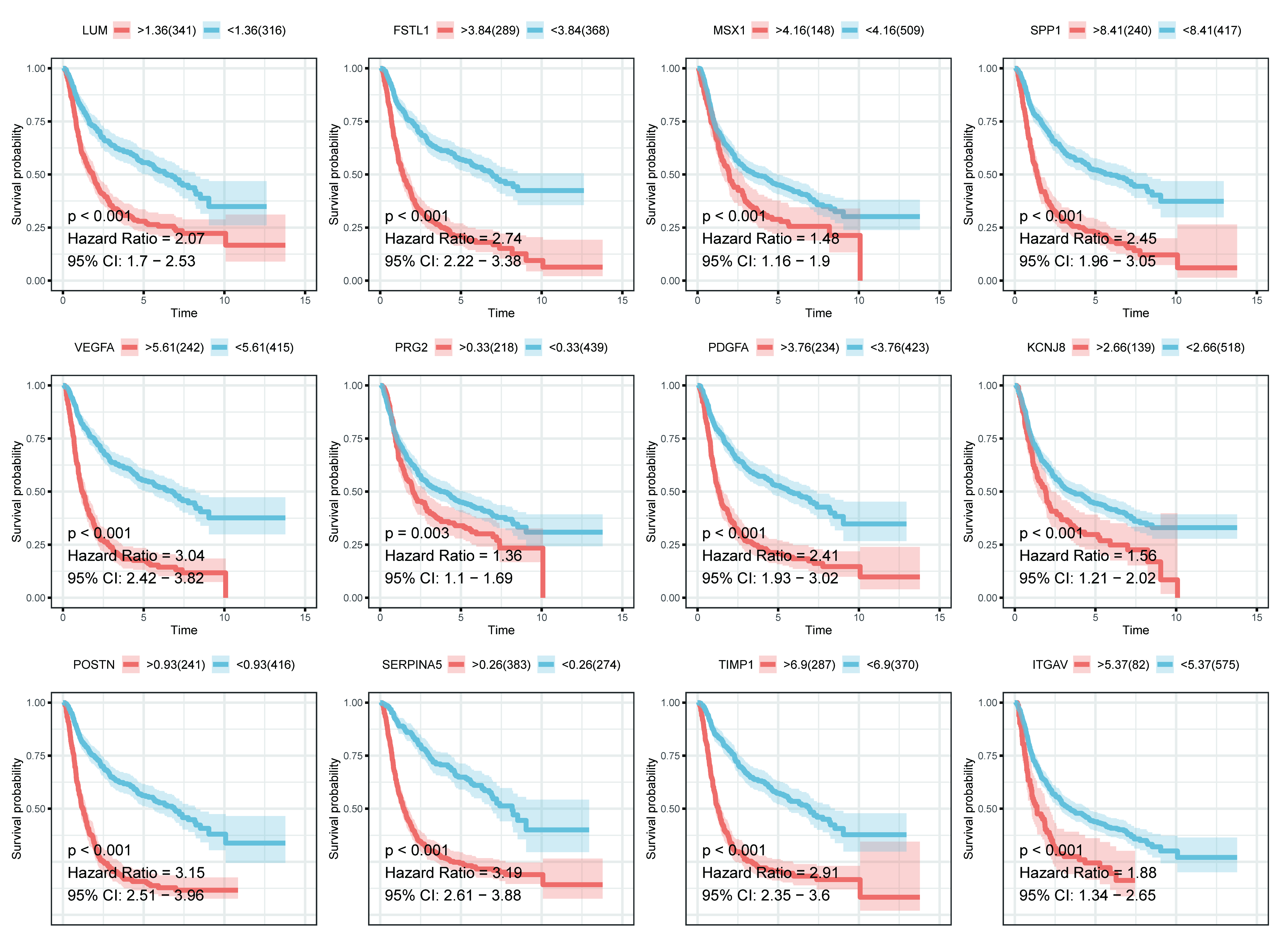

Supplement: Supplementary file 3 [file Image4.TIF]

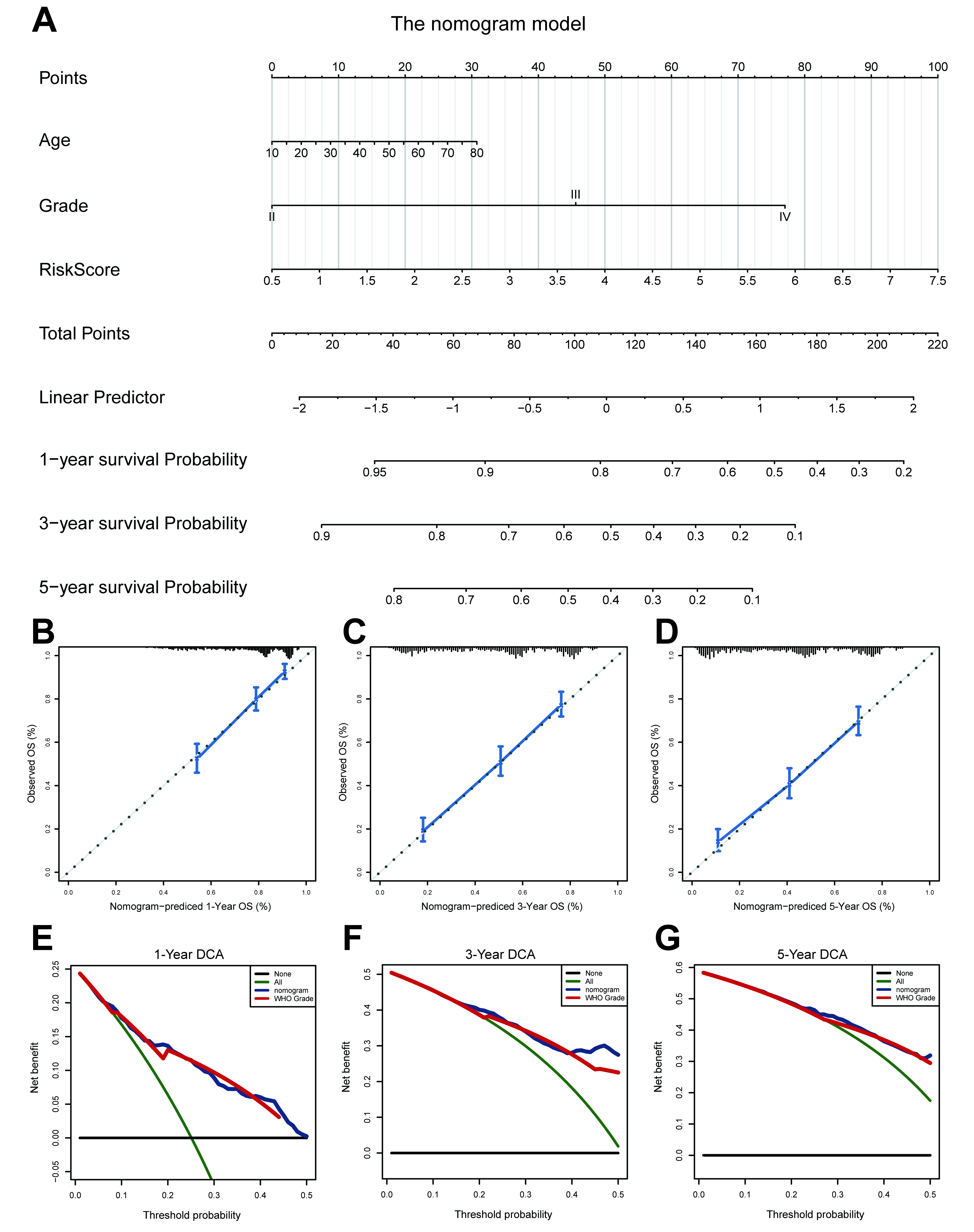

Supplement: Supplementary file 4 [file Image9.TIF]

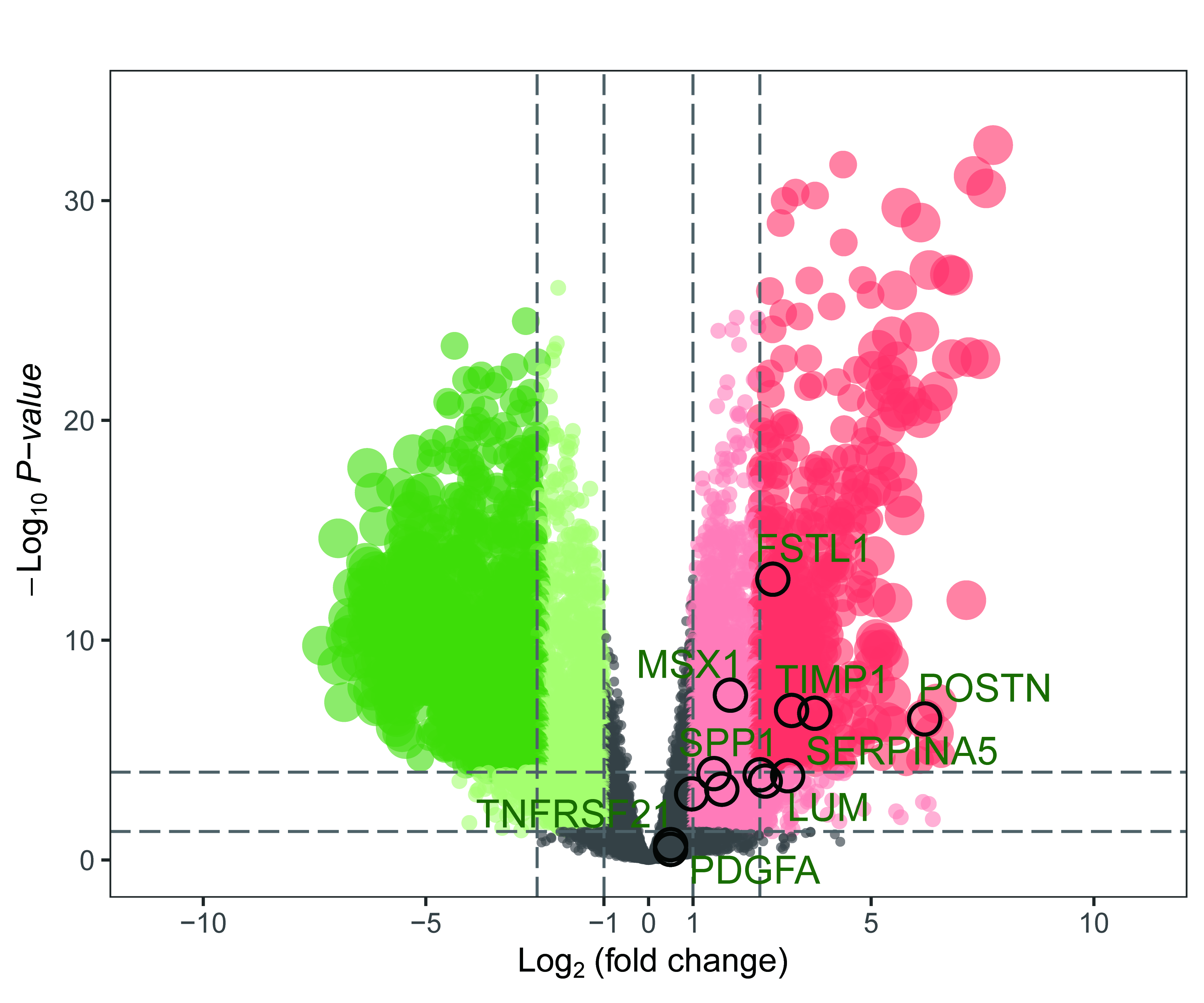

Supplement: Supplementary file 5 [file Image2.TIF]

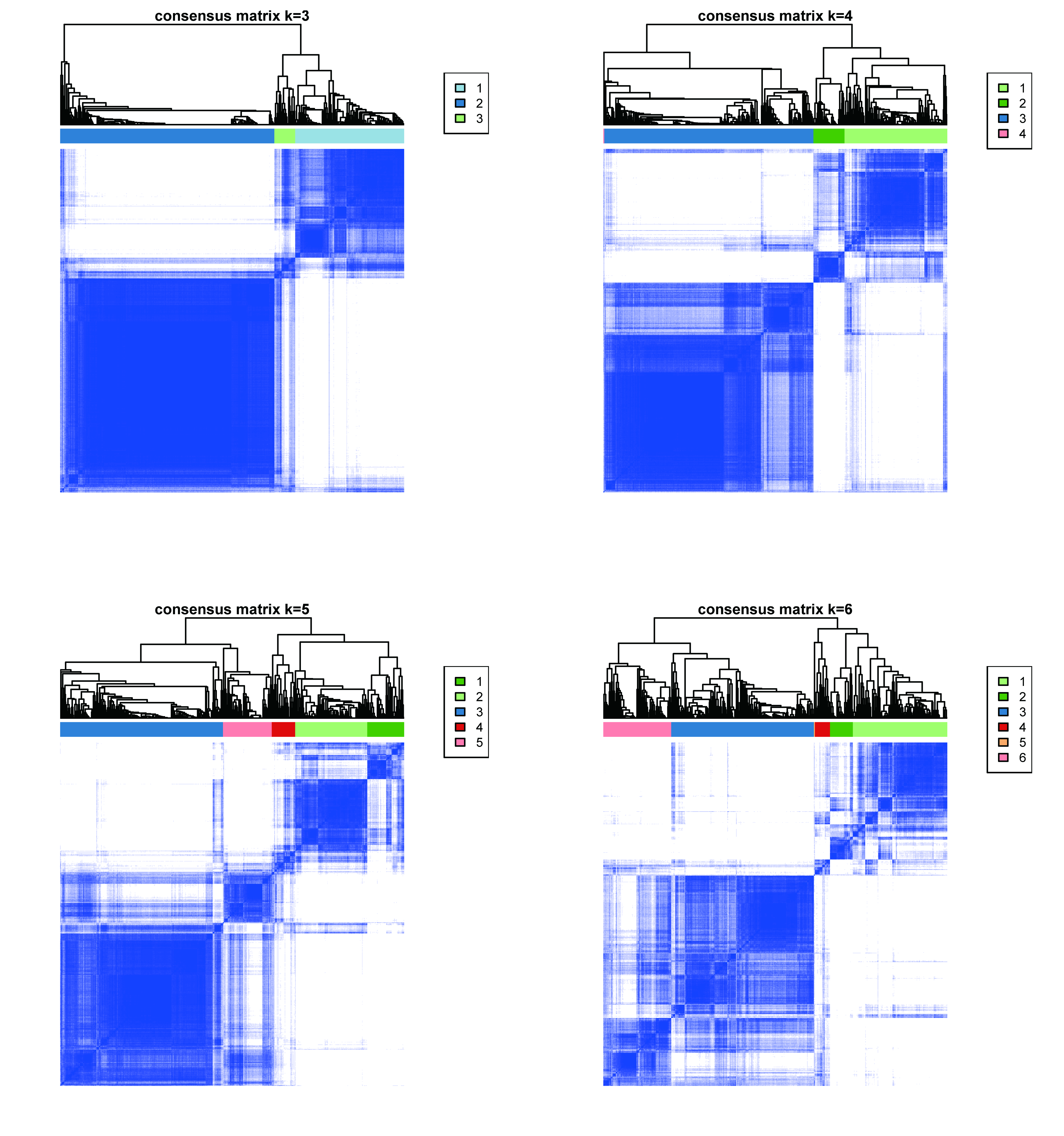

Supplement: Supplementary file 6 [file Image1.TIF]

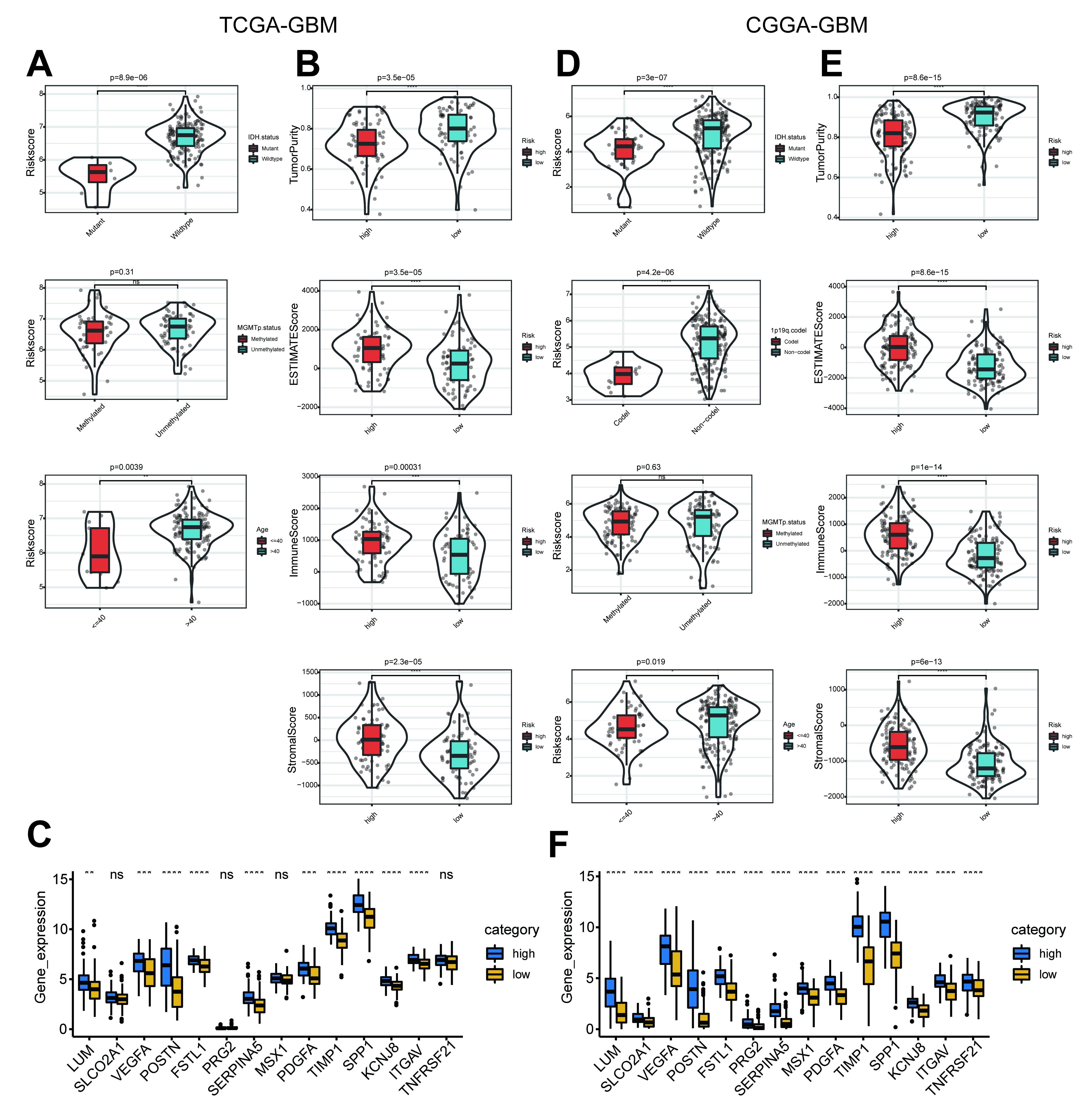

Supplement: Supplementary file 7 [file Image7.TIF]

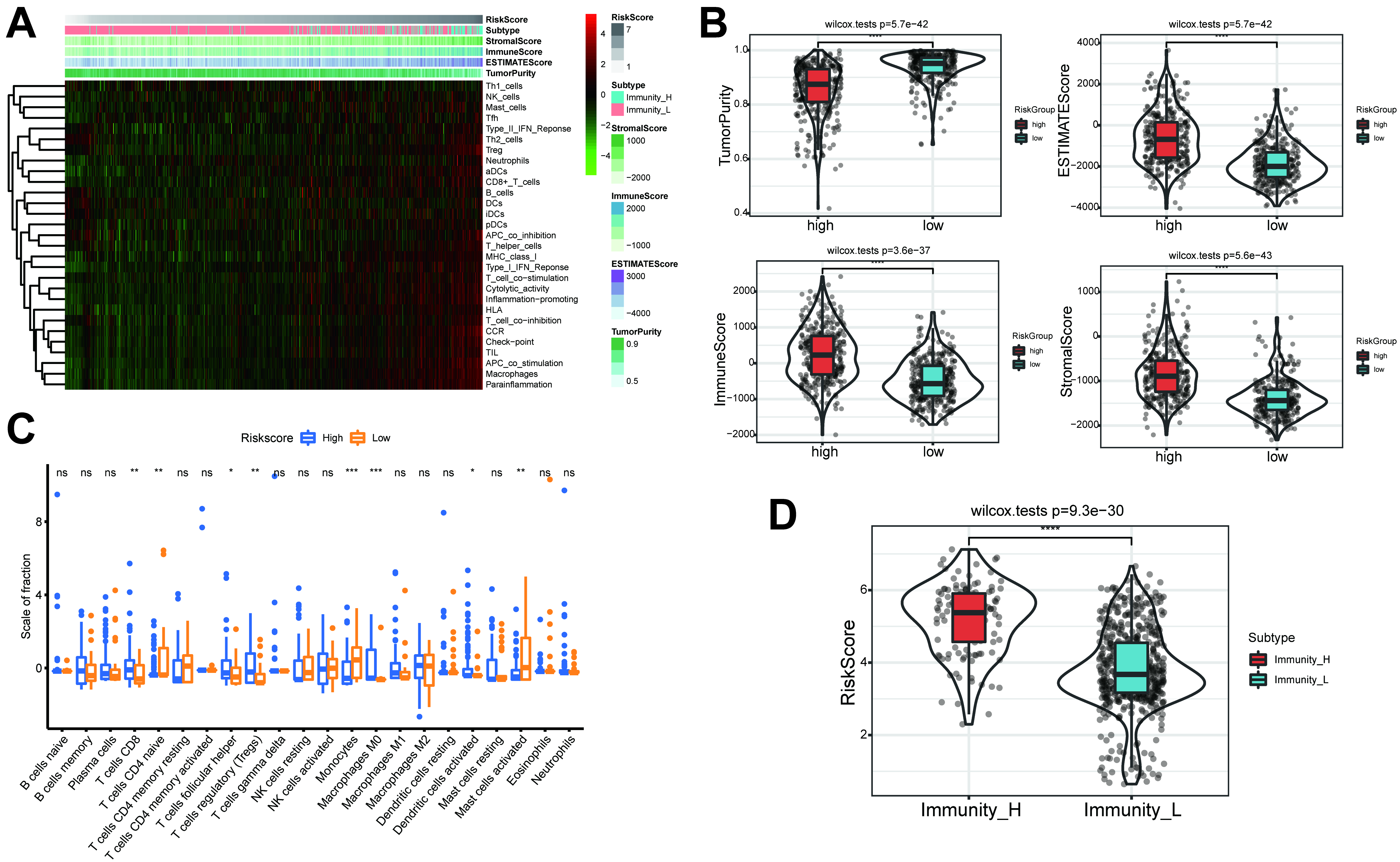

Supplement: Supplementary file 8 [file Image8.TIF]

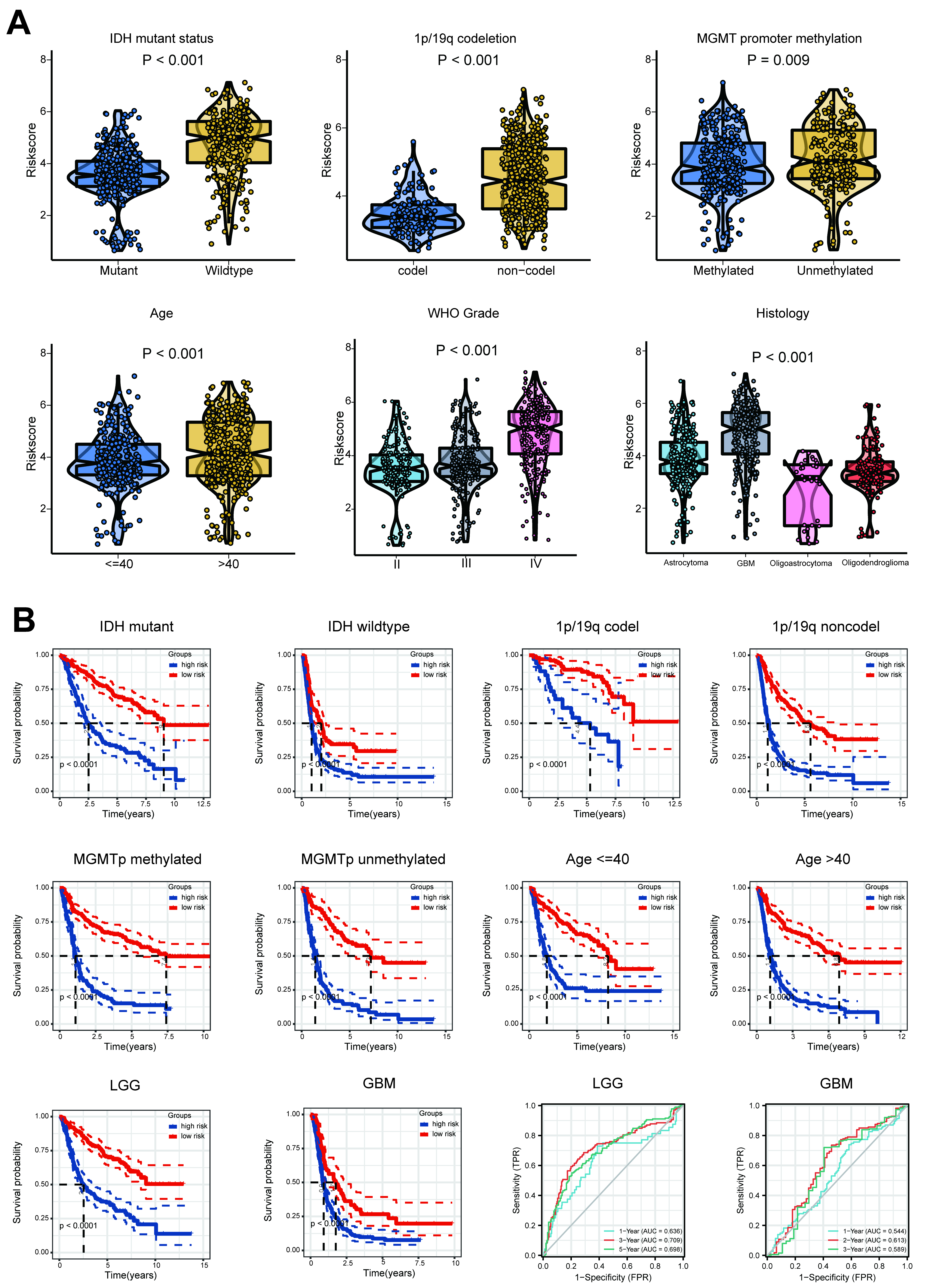

Supplement: Supplementary file 9 [file Image5.TIF]
