# Supplementary material for: Construction and validation of an angiogenesis-related gene expression signature associated with clinical outcome and tumor immune microenvironment in glioma
Source: Front Genet. 2022 Aug 11;13:934683. doi: 10.3389/fgene.2022.934683 (PMC9403517; doi:10.3389/fgene.2022.934683)
Supplement: Supplementary file 10 [file DataSheet1.docx]

**Supplementary Table 1. Characteristics of patients in TCGA cohort and CGGA cohort**

| Feature | TCGA cohort (n=663) | CGGA cohort (n=693) |
| --- | --- | --- |
| age |  |  |
| ≤40 | 265 | 291 |
| >40 | 398 | 401 |
| Grade |  |  |
| II | 248 | 188 |
| III | 262 | 255 |
| IV | 153 | 249 |
| Histology |  |  |
| Astrocytoma | 192 | 271 |
| Oligodendroglioma | 191 | 142 |
| Oligoastrocytoma | 127 | 31 |
| GBM | 153 | 249 |
| IDH-mutation status |  |  |
| Mutant | 422 | 356 |
| Wildtype | 234 | 286 |
| 1p/19q codeletion |  |  |
| Codel | 168 | 145 |
| Non-codel | 487 | 478 |
| MGMT promoter methylation |  |  |
| Methylated | 473 | 315 |
| Unmethylated | 159 | 227 |

**Supplementary Figure legends**

**Supplementary Figure S1.** The consensus matrices plot exhibits the clusters at *k* = 3, 4, 5, 6.

**Supplementary Figure S2.** Differential analysis between GBM and normal tissues. (adj. p-value < 0.05 and |log2(FC)| >1).

**Supplementary Figure S3.** Survival analysis of risk genes in the TCGA database.

**Supplementary Figure S4.** Survival analysis of risk genes in the CGGA database.

**Supplementary Figure S5.** (A) Relationship between risk score and each clinicopathological feature (*IDH*-mutant status, 1p/19q codeletion, MGMT promoter methylation, age, WHO grade, and histology). (B) KM analyses of patients in the CGGA dataset stratified by *IDH*-mutant status, 1p/19q codeletion, MGMT promoter methylation, age, and WHO grade in the CGGA cohort. ROC curve analysis of the risk model in predicting 1-, 3- and 5-year OS in the CGGA-LGG cohort and 1-, 2- and 3-year OS in the CGGA-GBM cohort.

**Supplementary Figure S6.** Clinical information analyses of LGG subgroup. (A, D) The relationship between the risk score and each clinicopathological feature (*IDH*-mutant status, 1p/19q codeletion, MGMT promoter methylation, age) in TCGA- and CGGA-LGG subgroup. (B, E) Tumor purity, ESTIMATE, immune and stromal scores in high- and low-risk groups in TCGA- and CGGA-LGG subgroup. (C, F) The expression differences of 14 ARGs between high and low-risk groups in TCGA- and CGGA-LGG subgroup.

**Supplementary Figure S7.** Clinical-information analyses of the GBM subgroup. (A, D) Relationship between risk score and each clinicopathological characteristic (*IDH*-mutant status, MGMT promoter methylation, age) in the TCGA- and CGGA-GBM subgroups. (B, E) Tumor purity and ESTIMATE, immune, and stromal scores in the high- and low-risk groups in the TCGA- and CGGA-GBM subgroups. (C, F) Expression differences of 14 ARGs between the high and low-risk groups in the TCGA- and CGGA-GBM subgroups.

**Supplementary Figure S8.** Relationship between risk signature and TIME in the CGGA database. (A) Heatmap of risk score and the two immunity subtypes based on ssGSEA scores for 29 immune gene sets in the CGGA database. (B) Comparison of tumor purity and of ESTIMATE, immune, and stromal scores between the high- and low-risk groups in the CGGA database. (C) Association between 22 tumor-infiltrating immune cells and the risk signature. (D) Comparison of risk score between the immunity-high and immunity-low subtypes in the CGGA database.

**Supplementary Figure S9.** Construction and validation of the nomogram to predict OS in glioma patients. (A) The nomogram was established using age, WHO grade, and the ARG risk signature in the CGGA cohort. (B–D) Calibration curve of the nomogram for predicting the probability of OS at 1, 3, and 5 years in the CGGA cohort. (E–G) DCA of the OS-related nomogram at 1, 3, and 5 years in the CGGA cohort.
